# Carcinomas assemble a filamentous CXCL12–keratin-19 coating that suppresses T cell–mediated immune attack

**DOI:** 10.1073/pnas.2119463119

**Published:** 2022-01-19

**Authors:** Zhikai Wang, Philip Moresco, Ran Yan, Jiayun Li, Ya Gao, Daniele Biasci, Min Yao, Jordan Pearson, Jaclyn F. Hechtman, Tobias Janowitz, Raza M. Zaidi, Matthew J. Weiss, Douglas T. Fearon

**Affiliations:** ^a^Cancer Center, Cold Spring Harbor Laboratory, Cold Spring Harbor, NY 11724;; ^b^Graduate Program in Genetics, Stony Brook University, Stony Brook, NY 11794;; ^c^Medical Scientist Training Program, Stony Brook University Renaissance School of Medicine, Stony Brook University, Stony Brook, NY 11794;; ^d^School of Biological Sciences, Cold Spring Harbor Laboratory, Cold Spring Harbor, NY 11724;; ^e^Cancer Research UK Cambridge Institute, University of Cambridge, Cambridge CB2 0RE, United Kingdom;; ^f^Department of Pathology, Memorial Sloan Kettering Cancer Center, New York, NY 10065;; ^g^Northwell Health Cancer Institute, Northwell Health, Lake Success, NY 11042;; ^h^Donald and Barbara Zucker School of Medicine at Hofstra/Northwell, Northwell Health, Hempstead, NY 11549;; ^i^Meyer Cancer Center, Weill Cornell Medicine, New York, NY 10065

**Keywords:** CXCL12, keratin-19, transglutaminase-2, T cells, cancer immunology

## Abstract

Cancer immunotherapy frequently fails because most carcinomas have few T cells, suggesting that cancers can suppress T cell infiltration. Here, we show that cancer cells of human pancreatic ductal adenocarcinoma (PDA), colorectal cancer, and breast cancer are coated with transglutaminase-2 (TGM2)–dependent covalent CXCL12–keratin-19 (KRT19) heterodimers that are organized as filamentous networks. Since a dimeric form of CXCL12 suppresses the motility of human T cells, we determined whether this polymeric CXCL12–KRT19 coating mediated T cell exclusion. Mouse tumors containing control PDA cells exhibited the CXCL12–KRT19 coating, excluded T cells, and did not respond to treatment with anti–PD-1 antibody. Tumors containing PDA cells not expressing either KRT19 or TGM2 lacked the CXCL12–KRT19 coating, were infiltrated with activated CD8^+^ T cells, and growth was suppressed with anti–PD-1 antibody treatment. Thus, carcinomas assemble a CXCL12–KRT19 coating to evade cancer immune attack.

## Significance

Carcinomas resist immunotherapy because T cells are absent from nests of cancer cells. The chemokine/chemokine receptor system, which regulates the migration of immune cells, is a candidate for this impaired intratumoral accumulation of T cells. Cancer cells in human pancreatic, colorectal, and breast cancers are coated with the chemokine CXCL12 in the form of covalent heterodimers with keratin-19. This CXCL12 coating was investigated using a mouse model of pancreatic cancer that replicates the immunological characteristics of human cancer. Mouse pancreatic cancer cells without the CXCL12 coating formed tumors that did not exclude T cells and responded to anti–PD-1 antibody treatment. Thus, the ability of cancer cells to coat themselves with CXCL12 may contribute to resistance to immunotherapy.

Contemporary cancer immunotherapy is based on studies showing that carcinomas can escape immune control by expressing membrane proteins, like PD-L1, that engage inhibitory receptors on T cells (1). However, since most carcinomas have relatively few infiltrating T cells (2), antagonists of these T cell checkpoints frequently fail. Therefore, an overarching immune resistance mechanism of cancer cells would be to restrict the influx of T cells. Although mechanisms have been proposed for how cancer cells alter the tumor microenvironment (TME) to limit the accumulation of intratumoral T cells (3–5), an immunological perspective would also consider a potential role for the chemokine/chemokine receptor system that regulates the trafficking of immune cells (6). The relevance of this immunological view was suggested by preclinical and clinical experiments showing that administering the bicyclam CXCR4 inhibitor AMD3100 to mice and patients with T cell–excluding carcinomas, microsatellite-stable (MSS) pancreatic ductal adenocarcinoma (PDA), and colorectal cancer (CRC) enhanced the intratumoral accumulation of activated T cells (7, 8). The additional observation that cancer cells in these tumors appeared to be “coated” with CXCL12, the ligand for CXCR4, suggested that the cancer cells have specifically adapted to the chemokine/chemokine receptor system. Understanding how cancer cells attach CXCL12 to their surfaces, therefore, may identify a pathway that enables tumors to suppress the infiltration of T cells and evade T cell–mediated immune attack.

## Results

### The Covalent CXCL12–Keratin-19 Heterodimeric “Coating” of Human PDA, CRC, and Breast Cancer Cells.

**Fig. 1.**
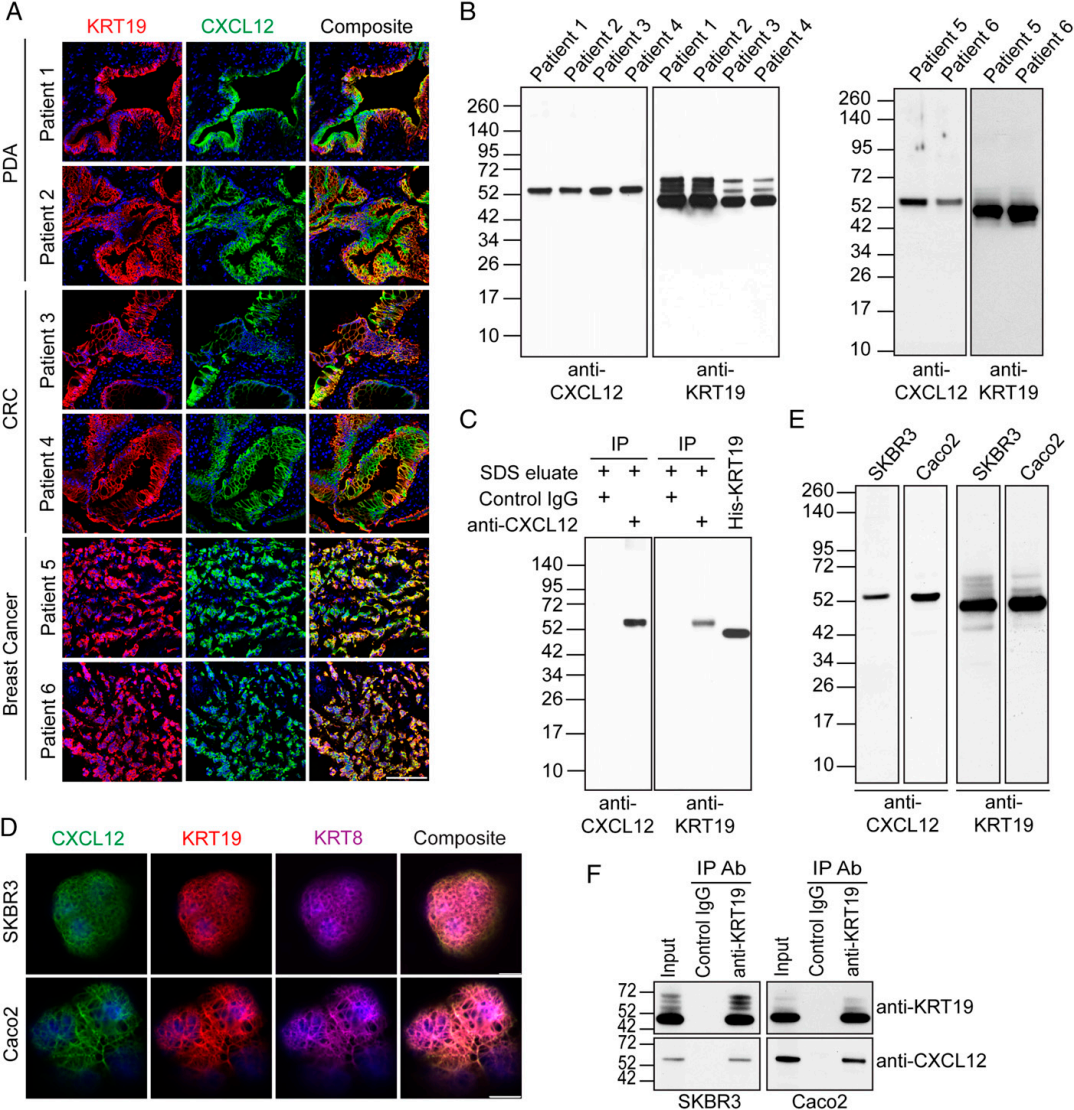
The CXCL12–KRT19 coating of cancer cells in human adenocarcinomas. (*A*) Sections of freshly resected human PDA, CRC, and breast cancers, respectively, were stained with fluorochrome-conjugated antibodies to KRT19 to reveal cancer cells and to CXCL12. (Scale bar, 100 µm.) (*B*) SDS eluates of the six tumors were subjected to SDS-PAGE and immunoblotting with anti-CXCL12 or anti-KRT19 antibodies. (*C*) The proteins that were immunoprecipitated by control IgG or anti-CXCL12 antibody from the SDS eluates of the human PDA tumors were subjected to SDS-PAGE and immunoblotting with anti-CXCL12 or anti-KRT19 antibodies. (*D*) The human breast cancer and CRC cell lines SKBR3 and Caco2, respectively, were stained with fluorochrome-conjugated antibodies to KRT19, KRT8, and CXCL12 prior to fixation and permeabilization. Confocal images of representative cells are shown. (Scale bar, 10 µm.) (*E*) SDS eluates of SKBR3 and Caco2 cells were subjected to SDS-PAGE and immunoblotting with antibodies to CXCL12 or KRT19. (*F*) The detergent lysates of SKBR3 and Caco2 cells were immunoprecipitated by control IgG or anti-KRT19 antibody and subjected to SDS-PAGE and immunoblotting with anti-CXCL12 and anti-KRT19 antibodies. Ab, antibody.

Frozen sections of surgically resected MSS PDA, MSS CRC, and breast cancer were stained with fluorochrome-conjugated antibodies to CXCL12 and keratin-19 KRT19. The KRT19^+^ cancer cells were specifically stained with anti-CXCL12 antibody (Fig. 1*A*). We sequentially extracted the tumor specimens with Nonidet P-40, sodium deoxycholate (DOC), DNase, and sodium dodecyl sulfate (SDS) and analyzed the lysates by SDS-polyacrylamide gel electrophoresis (SDS-PAGE) and immunoblotting with anti-KRT19 and anti-CXCL12 antibodies. CXCL12 was detected only in the SDS eluates of all tumors and only with an apparent molecular weight of ∼52 kDa, instead of its native molecular weight of 9 to 11 kDa (Fig. 1*B* and *SI Appendix*, Fig. S1). KRT19 also was released by SDS treatment of the tumors, as expected for a component of intermediate filaments (IF) (Fig. 1*B*). In addition to the ∼44-kDa major band corresponding to the native KRT19, there were several higher molecular weight bands, one of which comigrated with the ∼52-kDa form of CXCL12. Since this unusual form of CXCL12 required SDS for solubilization, and its apparent molecular weight was similar to a putative complex with KRT19, we subjected the SDS eluate from a PDA surgical specimen to immunoprecipitation (IP) with anti-CXCL12 antibody and analyzed the coprecipitated proteins by immunoblotting with anti-KRT19 antibody. A single band was revealed that comigrated with the high-molecular-weight form of CXCL12 (Fig. 1*C*). Thus, all detectable CXCL12 that was associated with cancer cells in six consecutive surgical samples of human PDA, CRC, and breast cancer, respectively, is covalently bound to KRT19.

KRT19, which lacks a signal peptide, is considered to be an intracellular component of the IF network of epithelial cells, but several human cancer cell lines externalize KRT19 (9, 10). Using cell surface biotinylation of intact SKBR3 breast cancer cells and Caco2 CRC cells, we demonstrated that the streptavidin (SA)-binding, biotinylated proteins from both cell lines included KRT19 and KRT8 (*SI Appendix*, Fig. S2*A*), a type II keratin protein that dimerizes with KRT19. The positive controls for known membrane proteins HER2 and cadherin-1 were biotinylated on SKBR3 cells and on Caco2 cells, respectively, and the negative control, the intracellular protein GAPDH, was not biotinylated in either cell line. Since these two cell lines externalized KRT8 and KRT19, and the fetal calf serum in which they were cultured contained CXCL12 (*SI Appendix*, Fig. S2*B*), we determined whether the CXCL12–KRT19 coating could be formed in vitro by these cancer cells. Confocal microscopy of cells stained with fluorochrome-conjugated antibodies to CXCL12, KRT19, and KRT8 showed that all three proteins colocalized in a higher-order network of discrete filaments covering the entire surfaces of the cells (Fig. 1*D* and *SI Appendix*, Fig. S2*C*). No cells staining only with anti-KRT19 or anti-CXCL12 antibody were observed. SDS-PAGE and immunoblotting of detergent lysates and anti-KRT19 antibody IPs of both cell lines revealed that this filamentous CXCL12–KRT19 coating was comprised of the CXCL12–KRT19 covalent heterodimer (Fig. 1 *E* and *F*). SKBR3 cancer cells were found to express *Cxcl12* mRNA (*SI Appendix*, Fig. S2*D*), which allowed an analysis of whether formation of the CXCL12–KRT19 heterodimer could occur in a cell-autonomous manner. Indeed, SKBR3 cancer cells cultured in serum-free medium stained with fluorochrome-conjugated antibodies to CXCL12 and KRT19 and had formed the CXCL12–KRT19 heterodimer (*SI Appendix*, Fig. S2 *E*–*G*).

### The Interactions among Recombinant KRT19, CXCL12, and Transglutaminase-2.

**Fig. 2.**
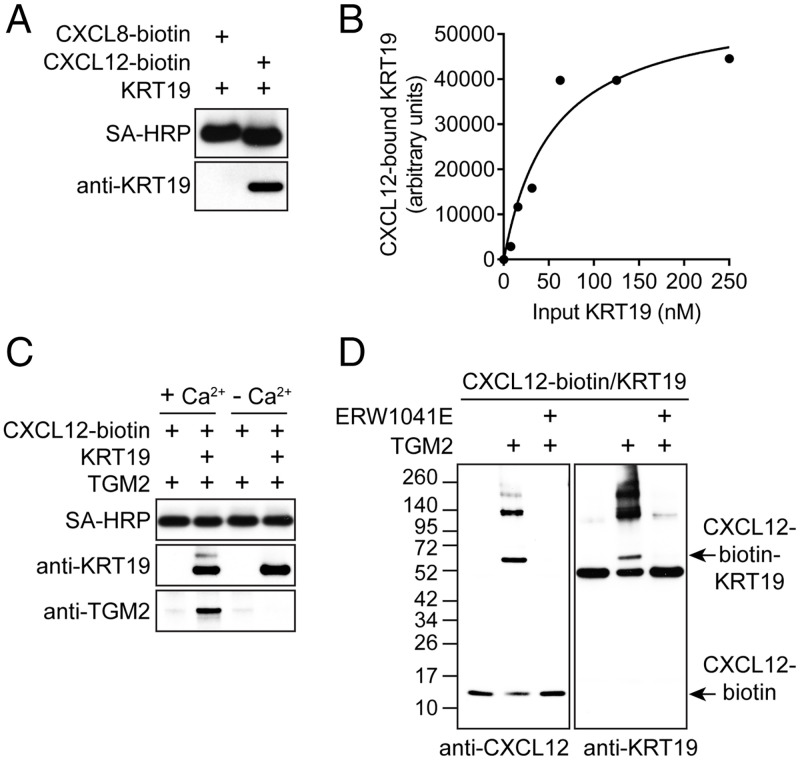
The interactions among CXCL12, KRT19, and TGM2. (*A*) Biotinylated human CXCL8 or CXCL12 was immobilized on SA beads, which were incubated with KRT19. The bead-bound proteins were eluted with SDS and subjected to SDS-PAGE and immunoblotting with SA-HRP or anti-KRT19 antibody. (*B*) SA beads bearing CXCL12–biotin were incubated with increasing concentrations of KRT19. Bound KRT19 was detected by SDS-PAGE and immunoblotting with anti-KRT19 antibody, and intensities of the KRT19 bands were measured. (*C*) CXCL12–biotin that was immobilized on SA beads was incubated at 4 °C with TGM2 in the absence or presence of KRT19 and in the absence or presence of 10 mM CaCl_2_. The bound proteins were eluted and subjected to SDS-PAGE and immunoblotting with SA-HRP and antibodies to KRT19 and TGM2. (*D*) SA beads bearing preformed complexes of CXCL12–biotin and KRT19 were incubated at 20 °C for 15 min with TGM2 in the absence or presence of the TGM2 inhibitor ERW1041E. The proteins were eluted from the beads and detected by SDS-PAGE and immunoblotting with antibodies to CXCL12 and KRT19.

That the SKBR3 and Caco2 cell lines form the CXCL12–KRT19 coating in the absence of the TME suggested that only three proteins were absolutely required for its formation: CXCL12 and KRT19, the two components of the heterodimer, and an enzyme that covalently cross-linked them. We selected transglutaminase-2 (TGM2), which forms isopeptide bonds between glutamines and ε-amino groups of lysines (11), as the candidate because it is expressed in human PDA, CRC, and breast cancers (12) and is present in lysates of the SKBR3 and Caco2 cell lines (*SI Appendix*, Fig. S3). First, to account for specificity of CXCL12 for KRT19 in the covalent heterodimers, we assessed whether the two proteins interacted in a noncovalent manner by incubating SA beads to which biotinylated CXCL12 had been attached with soluble KRT19. A specificity control was also performed with SA beads bearing biotinylated CXCL8. After washing the beads, the bound proteins were eluted and subjected to SDS-PAGE and immunoblotting with SA–horseradish peroxidase (HRP) and anti-KRT19 antibody. KRT19 bound to the CXCL12-coated beads but not beads coated with CXCL8 (Fig. 2*A*). When the amounts of KRT19 that were incubated with the CXCL12-bearing beads were varied, we observed a saturable reaction that reached half-maximal binding at ∼50 nM KRT19 (Fig. 2*B*). Second, TGM2 was also found to bind to the SA bead-immobilized CXCL12 at 4 °C but only in the presence of bound KRT19 and only in the presence of Ca^2+^ (Fig. 2*C*). Thus, KRT19 forms a ternary complex with CXCL12 and TGM2, with binding of the latter requiring its Ca^2+^-dependent, enzymatically active open conformation (11). Third, when a preformed, noncovalent complex of KRT19 and CXCL12 was incubated with TGM2 at 20 °C, a covalent CXCL12–KRT19 heterodimer was generated that resembled that which is associated with cancer cells (Fig. 2*D*). Inclusion of the TGM inhibitor ERW1041E blocked formation of the heterodimer (Fig. 2*D*), showing that the TGM activity of TGM2 is required. The additional, higher-molecular-weight CXCL12–KRT19 complexes formed with recombinant proteins (Fig. 2*D*) may indicate that cancer cells have additional constraints on the TGM2-mediated cross-linking of these proteins.

### Dimeric CXCL12 and a CXCR4 “Stop” Signal.

**Fig. 3.**
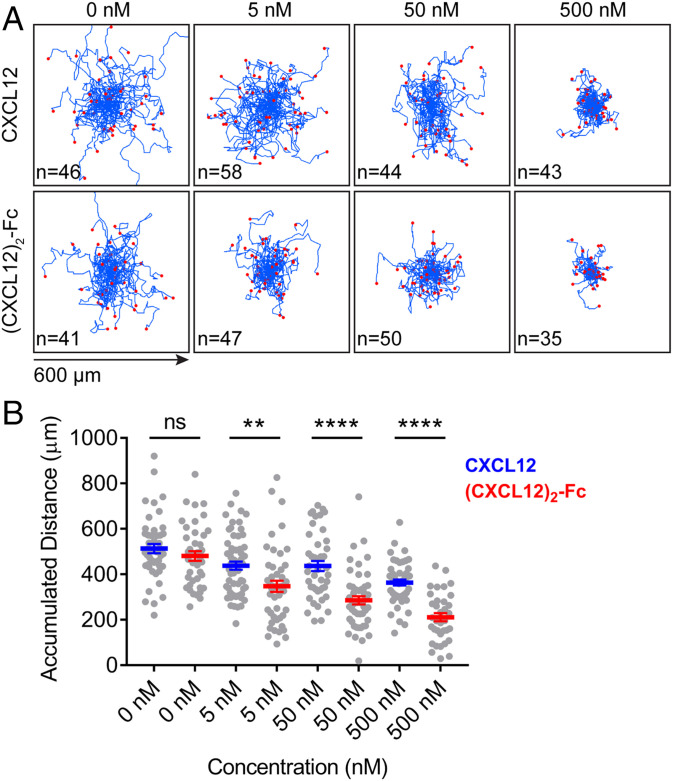
Motility of human T lymphoblastoid cells in response to monomeric and dimeric CXCL12. (*A*) Aggregated trajectories of the spontaneous motility of CCRF-HSB2 cells in the absence or presence of increasing concentrations of CXCL12 monomer or (CXCL12)_2_–Fc fusion protein were monitored by continuous live-cell imaging for 2 h. (*B*) The accumulated distance of each cell was calculated and plotted. Mean ± SEM; ns, not significant; **P < 0.01, ****P < 0.0001, Student’s *t* test.

Having found that the surfaces of cancer cells display CXCL12–KRT19 heterodimers that are polymerized by inclusion into filamentous networks, we wished to examine how ligation of CXCR4 by a multimeric form of CXCL12 would affect the motility of T cells. CXCR4 is multifunctional and, in addition to its mediating chemotaxis, can mediate chemorepulsion, inhibit the capacity of other chemokine receptors to stimulate the directed migration of immune cells, and, when ligated with dimeric CXCL12, inhibit chemotaxis to monomeric CXCL12 (8, 13–15). The absence of T cells in cancer cell nests suggests that the polymeric CXCL12–KRT19 coating of cancer cells may elicit a CXCR4 signal that affects random T cell motility. As a model for polymeric CXCL12, we prepared (CXCL12)_2_–Fc, a bivalent Fc fusion protein containing wild-type CXCL12, which bound to human T lymphoblastoid cells in a CXCR4-dependent manner (*SI Appendix*, Fig. S4). We tracked the spontaneous motility of individual human CCRF-HSB2 T lymphoblastoid cells in the absence or presence of monomeric CXCL12 and the (CXCL12)_2_–Fc fusion protein, respectively. Stimulation of CXCR4 by the dimeric form of CXCL12 significantly reduced the spontaneous motility of the T cells with respect to the effects of buffer alone or CXCL12 at equivalent concentrations (Fig. 3). Higher concentrations of CXCL12 modestly affected T cell motility, which may reflect its tendency to dimerize (15). Thus, cross-linking CXCR4 by dimeric CXCL12 may elicit a “stop” signal that differs from the chemotactic signal stimulated by monomeric CXCL12 but may be similar to that elicited by the polymeric form of CXCL12 that is distributed on the surfaces of cancer cells.

### The CXCL12–KRT19 Heterodimer of Mouse PDA Cells and the Exclusion of T Cells.

These experiments predicted that preventing expression by cancer cells of either KRT19 or TGM2 would impair formation of the CXCL12–KRT19 coating and allow T cells to accumulate within cancer cell nests. We demonstrated that the mouse PDA tumor was a suitable model for this analysis by showing that the KRT19^+^ cancer cells in subcutaneous (s/c) PDA tumors were coated with CXCL12, as assessed by imaging nonpermeabilized sections that had been stained with antibodies to KRT19 and CXCL12 (*SI Appendix*, Fig. S5 *A* and *B*). The specificity of the anti-CXCL12 antibody was demonstrated by the capacity of recombinant CXCL12 to block its staining of cancer cells (*SI Appendix*, Fig. S5*A*). The presence on the cell surface of CXCL12 and KRT19 was confirmed by flow cytometric analysis of single-cell suspensions of intact tumor cells (*SI Appendix*, Fig. S5*C*). Also, and most importantly for this analysis, the ∼52-kDa form of CXCL12 that was eluted from s/c PDA tumors was found by co-IP to be a complex with KRT19 (*SI Appendix*, Fig. S5 *D* and *E*). ERW1041E had no effect on the recovery of a preformed ∼52-kDa CXCL12–KRT19 heterodimer from PDA cells (*SI Appendix*, Fig. S5*F*), in contrast to its capacity to inhibit the formation of the CXCL12–KRT19 heterodimer in vitro (Fig. 2*D*).

**Fig. 4.**
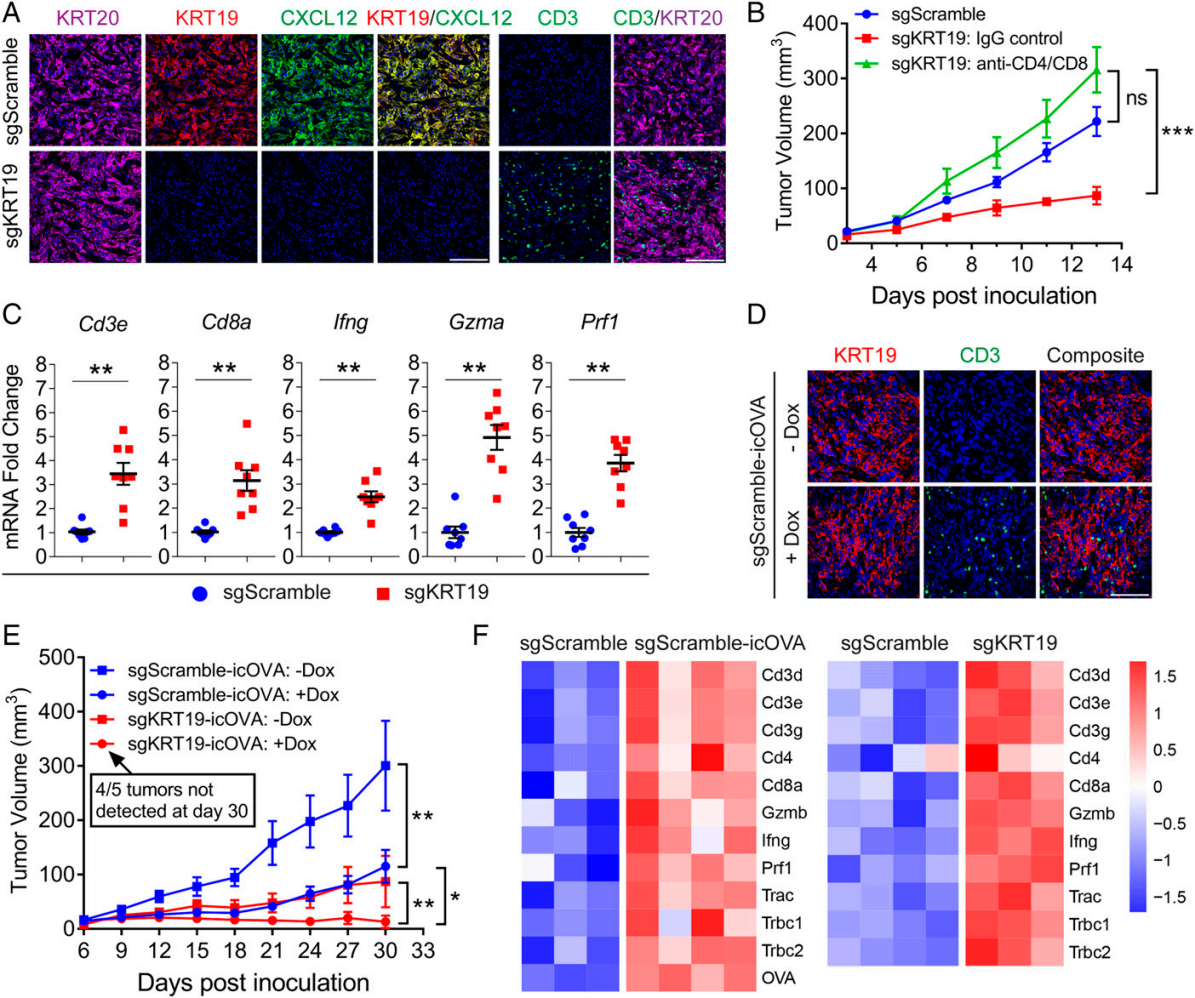
The immunological responses of tumors formed with *Krt19*-edited mouse PDA cells. (*A*) Sections of s/c tumors formed with sgScramble control PDA cells and sgKRT19-edited PDA cells were stained with fluorochrome-conjugated antibodies to KRT20, KRT19, CXCL12, and CD3. (Scale bars, 100 µm.) (*B*) Growth curves are shown of s/c tumors formed with sgScramble control PDA cells and sgKRT19-edited PDA cells in mice that were untreated or treated with either nonimmune IgG or T cell–depleting antibodies to CD4 and CD8; *n* = 5. (*C*) The mRNA levels of immune genes in tumors formed with sgKRT19-edited PDA cells were compared to those of tumors formed with sgScramble control PDA cells; *n* = 8. (*D*) Sections of s/c tumors formed with sgScramble control PDA cells without or with Dox-induced icOVA expression were stained with fluorochrome-conjugated antibodies to KRT19 and CD3. (Scale bar, 100 μm.) (*E*) Growth curves are shown of s/c tumors formed with sgScramble control PDA cells or sgKRT19-edited PDA cells without or with Dox-induced icOVA expression; *n* = 5; mean ± SEM; ns, not significant, *P < 0.05, **P < 0.01, ***P < 0.001; Student’s *t* test. (*F*) The heat map depicts the relative expression of immune genes as assessed by RNA-seq assay of individual s/c tumors formed with paired sgScramble control PDA cells with icOVA-expressing sgScramble control PDA cells and paired sgScramble control PDA cells with sgKRT19-edited PDA cells.

We generated s/c tumors with PDA cells in which the *Krt19* gene was intact or had been CRISPR/Cas9 edited (*SI Appendix*, Fig. S6 *A* and *B*). Cancer cells in tumors containing control, scrambled small guide (sgScramble) PDA cells demonstrated staining with fluorochrome-conjugated antibodies to KRT20, KRT19, and CXCL12, respectively, and CD3^+^ T cells were infrequent in the areas of the KRT20^+^ cancer cells. In contrast, s/c tumors formed with the *Krt19*-edited PDA cells did not stain with antibodies to either KRT19 or CXCL12, and intratumoral CD3^+^ T cells were frequent (Fig. 4*A* and *SI Appendix*, Fig. S6*C*). As a control for this role of KRT19, s/c tumors were formed with PDA cells in which *Krt18* had been edited and were observed to be stained with anti-CXCL12 antibody (*SI Appendix*, Fig. S6 *D* and *E*). Tumors also were formed by *Krt19*-edited PDA cells that had been rescued by expressing ectopic KRT19, and the cancer cells had regained the capacity to form the CXCL12–KRT19 coating and exclude T cells (*SI Appendix*, Fig. S7). PDA tumors lacking the CXCL12–KRT19 coating grew more slowly than sgScramble control tumors, and the slower growth was reversed by T cell depletion (Fig. 4*B*). This role for T cells in mediating the slower growth was supported by finding enhanced expression of genes that characterize activated cytotoxic T cells (CTLs) in tumors lacking the CXCL12–KRT19 coating (Fig. 4*C*).

Human microsatellite-instable (MSIhigh) CRC and mouse tumor models of mismatch repair deficiency are hypermutated, contain many potential neoantigens, and typically are infiltrated with T cells (16). Yet, human MSIhigh PDA tumors exhibited staining with anti-CXCL12 antibody that was equivalent to that of MSS PDA (*SI Appendix*, Fig. S8). This finding suggests that the CXCL12–KRT19 coating either may not be biologically relevant in highly immunogenic cancers or, alternatively, that immunogenicity and the CXCL12–KRT19 coating independently affect the interaction of cancer cells with the immune system. We modeled the heightened immunogenicity of neoantigens resulting from the frameshift mutations of MSIhigh PDA tumors by introducing an exogenous antigen (17). We expressed doxycycline (Dox)-inducible intracytoplasmic ovalbumin (icOVA) in mouse PDA cells that did or did not express KRT19 and formed s/c tumors with these PDA cells in the presence or absence of continuous Dox treatment of mice. The tumors having Dox-induced icOVA expression by KRT19^+^ PDA cells, which had the CXCL12–KRT19 coating, were infiltrated with CD3^+^ T cells (Fig. 4*D*). The growth rates of the tumors formed by these icOVA-expressing KRT19^+^ PDA cells were slower than those of tumors comprised of the non–icOVA-expressing KRT19^+^ PDA cells and similar to those of s/c tumors formed by *Krt19*-edited PDA cells not expressing icOVA (Fig. 4*E*). RNA-sequencing (RNA-seq) analysis indicated that the enhanced immune responses occurring in tumors formed with PDA cells expressing icOVA or lacking the CXCL12–KRT19 coating were similar (Fig. 4*F*). Tumors comprised of *Krt19*-edited PDA cells that combined icOVA expression with an absence of the CXCL12–KRT19 coating exhibited a further reduction of tumor growth rate, and by day 30, four of five tumors were no longer detectable (Fig. 4*E*). Therefore, the CXCL12–KRT19 coating of cancer cells mediates immune suppression even in a highly immunogenic PDA tumor with spontaneous T cell infiltration.

**Fig. 5.**
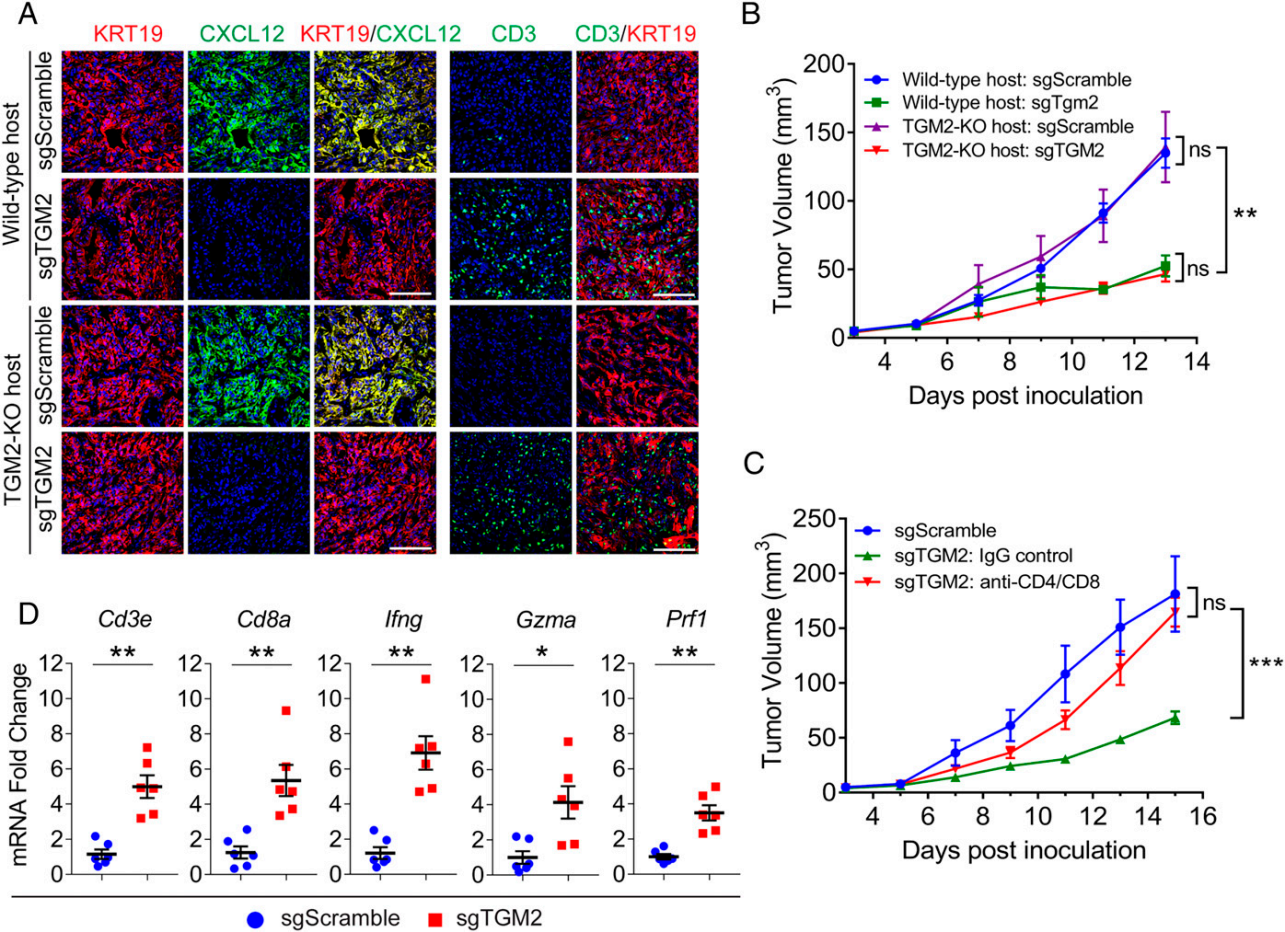
The immunological responses of tumors formed with *Tgm2*-edited mouse PDA cells. sgScramble control or sgTGM2-edited PDA cells were inoculated s/c in wild-type hosts or TGM2-deficient hosts. (*A*) Sections of each of the four tumor types were stained with fluorochrome-conjugated antibodies to KRT19, CXCL12, and CD3. Representative images are shown. (Scale bars, 100 µm.) (*B*) The growth curves of each of the four tumor types are shown; *n* = 5. (*C*) Growth curves are shown of tumors formed with sgScramble control PDA cells and sgTGM2-edited PDA cells, respectively, in wild-type hosts that were untreated or treated with nonimmune IgG or T cell–depleting antibodies to CD4 and CD8; *n* = 5. (*D*) The mRNA levels of immune genes in tumors formed with sgTGM2-edited PDA cells in wild-type hosts were compared to those of tumors formed with sgScramble control PDA cells; *n* = 6; mean ± SEM; ns, not significant, *P < 0.05, **P < 0.01, ***P < 0.001; Student’s *t* test.

The role for TGM2 in formation of the CXCL12–KRT19 coating on mouse PDA cells was assessed with s/c tumors having PDA cells in which the *Tgm2* gene had been CRISPR/Cas9 edited (*SI Appendix*, Fig. S9). The tumors were formed in both wild-type mice and mice lacking expression of TGM2 (TGM2 knockout [TGM2-KO]) to determine whether the source of TGM2 was important. Control PDA cells expressing TGM2 had the CXCL12–KRT19 coating in tumors from wild-type and TGM2-KO mice, and CD3^+^ T cells were rarely observed. In contrast, tumors that contained PDA cells lacking expression of TGM2 had diminished appearance of the CXCL12–KRT19 coating in both wild-type and TGM2-KO mice, indicating that TGM2 must be expressed by the cancer cell (Fig. 5*A* and *SI Appendix*, Fig. S10*A*). Tumors with diminished formation of the CXCL12–KRT19 coating showed increased numbers of infiltrating CD3^+^ T cells (Fig. 5*A*) and slower growth rates than the tumors formed with PDA cells expressing TGM2 in both types of hosts (Fig. 5*B*). That T cells mediated this growth restriction was demonstrated by the effects of their depletion (Fig. 5*C*) and by the elevated mRNA levels of genes in the tumors that indicate the presence of CTLs (Fig. 5*D*). Scanning entire cross-sections of the s/c tumors formed with PDA cells lacking expression of TGM2 from either wild-type mice or TGM2-KO mice revealed patchy areas of residual staining with anti-CXCL12 antibody (*SI Appendix*, Fig. S10*A*). This residual staining with anti-CXCL12 antibody reflected decreased amounts of the CXCL12–KRT19 heterodimer (*SI Appendix*, Fig. S10*B*), despite an absence of TGM2 protein in the tumors formed with PDA cells lacking TGM2 expression in the TGM2-KO host (*SI Appendix*, Fig. S10*C*). Since the PDA cells did not express other members of the TGM family (*SI Appendix*, Fig. S10*D*), this residual CXCL12–KRT19 coating may have been generated by the clotting factor F13a1 that was present in all PDA tumors (*SI Appendix*, Fig. S10*E*) and may be produced by infiltrating myelomonocytic cells. However, this residual CXCL12–KRT19 coating is not as immunologically relevant as that generated by TGM2. This role for TGM2 was supported by finding that formation of the CXCL12–KRT19 coating and the exclusion of T cells in the s/c tumors were rescued by expression of ectopic TGM2 in the *Tgm2*-edited PDA cells (*SI Appendix*, Fig. S11).

### The CXCL12–KRT19 Coating of Mouse PDA Cells and Resistance to Blocking the PD-1 T Cell Checkpoint.

**Fig. 6.**
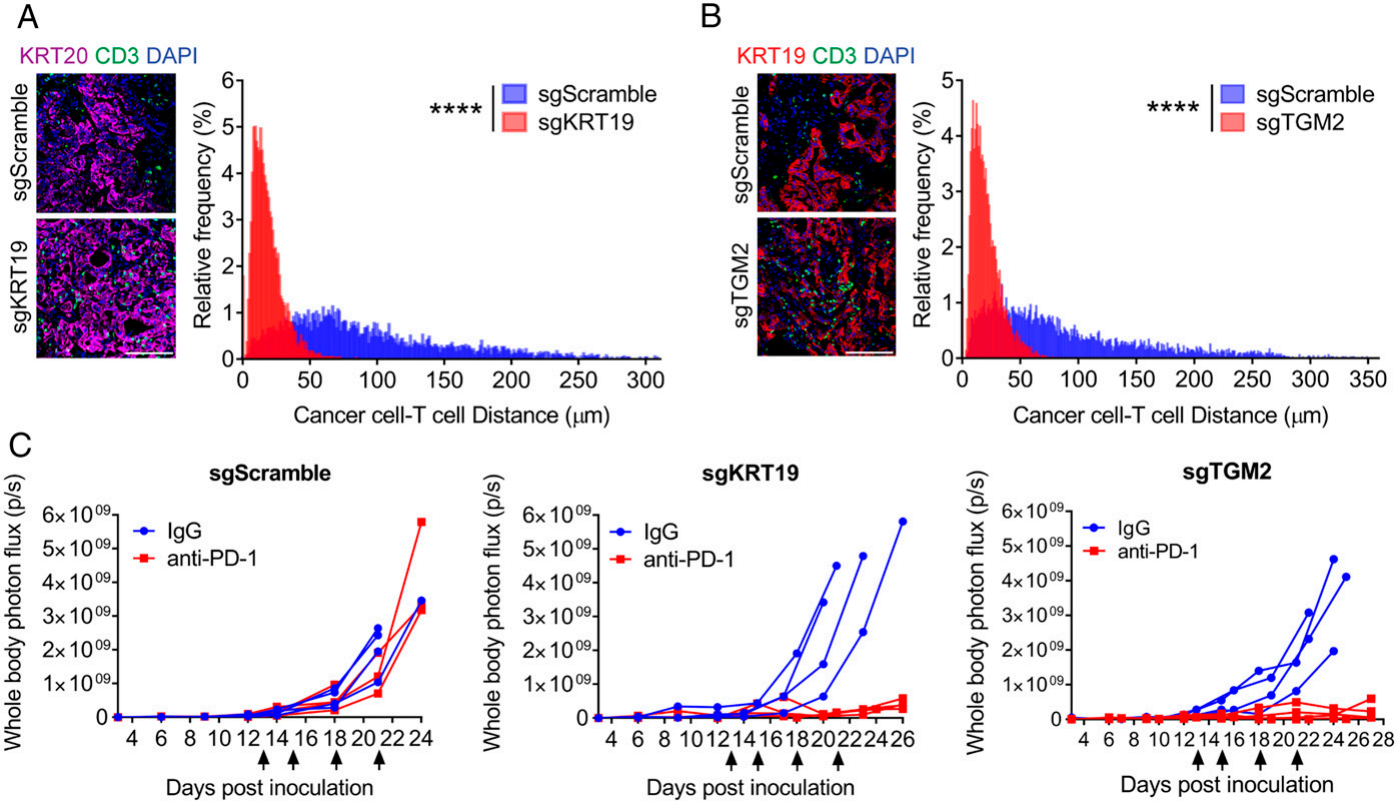
Responses of hepatic metastases formed with control, *Krt19*-edited, or *Tgm2*-edited PDA cells to treatment with anti–PD-1 antibody. (*A* and *B*) Sections of hepatic metastases formed with luciferase-expressing sgScramble control PDA cells, sgKRT19-edited (*A*), or sgTGM2-edited (*B*) PDA cells were stained with fluorochrome-conjugated antibodies to KRT20 or KRT19 to reveal cancer cells and with CD3 to reveal T cells. The distance of each cancer cell from the nearest T cell was calculated for entire cross-sections, and the distance distribution of the sgScramble control tumors and their matching sgKRT19 or sgTGM2 tumors were plotted. (Scale bars, 100 µm.) More than 4,000 cancer cells from two to seven tumor sections of each group were analyzed; ****P < 0.0001, Kolmogorov–Smirnov test. (*C*) Mice bearing hepatic metastases formed with these luciferase-expressing PDA cells were treated with nonimmune IgG or anti–PD-1 IgG (arrows), and growth of the metastases was measured by bioluminescent imaging.

We examined the role of the CXCL12–KRT19 coating of cancer cells in mediating the resistance of mouse PDA tumors to inhibition of the PD-1 T cell checkpoint (7). We chose a model of hepatic metastases rather than orthotopic tumors since metastatic disease represents a major therapeutic challenge in PDA patients, and the immunological microenvironment of the hepatic metastasis model is similar to that of the orthotopic PDA tumor (*SI Appendix*, Fig. S12). Hepatic metastases were established by portal vein injection of luciferase-expressing PDA cells that expressed both KRT19 and TGM2 or PDA cells in which the *Krt19* or *Tgm2* genes had been CRISPR/Cas9 edited. The metastases formed with PDA cells expressing both KRT19 and TGM2 exhibited the CXCL12–KRT19 coating, and cancer cell nests excluded T cells. Metastases comprised of either Krt19- or Tgm2-edited PDA cells lacked the CXCL12–KRT19 coating and had greater numbers of CD3^+^ T cells (*SI Appendix*, Fig. S13 *A* and *B*). Quantitating the spatial relationship between the intratumoral T cells and cancer cells across the entire tumor areas showed that the increased numbers of T cells significantly decreased the distances separating each cancer cell from the nearest T cell (Fig. 6 *A* and *B*). This altered intratumoral distribution of T cells was associated with elevated mRNA levels of genes indicating the presence of CTLs (*SI Appendix*, Fig. S13 *C* and *D*). Mice bearing these three types of hepatic metastases were administered intraperitoneal injections of control rat immunoglobulin G (IgG) or rat anti–PD-1 IgG at 13, 15, 18, and 21 d after portal vein injection of cancer cells, after which treatment was discontinued because of the occurrence of mouse anti-rat IgG antibodies (*SI Appendix*, Fig. S14). Inhibiting PD-1 had no effect on the growth of the metastatic lesions formed with control PDA cells but prevented expansion of metastases formed with PDA cells lacking expression of KRT19 or TGM2 (Fig. 6*C*). Similar contrasting responses to administration of anti–PD-1 antibody to mice bearing the three types of s/c tumors were observed (*SI Appendix*, Fig. S15*A*), and inhibition of this immune checkpoint markedly enhanced expression of genes characteristic of CTLs only in the tumors that lacked the CXCL12–KRT19 coating (*SI Appendix*, Fig. S15*B*). Thus, the lack of CXCL12–KRT19 coating of PDA cells is directly responsible for the enhanced intratumoral accumulation of activated CD8^+^ T cells and sensitivity to treatment with anti–PD-1 antibody.

## Discussion

To support the proposal that cancer cells of carcinomas interact with the chemokine/chemokine receptor system to escape immune attack, we defined how they assemble a TGM2-dependent, filamentous coating of CXCL12–KRT19 heterodimers that restricts T cell motility and suppresses the intratumoral accumulation of T cells. The remarkably specific association of CXCL12 with KRT19 on cancer cells in human and mouse tumors and on human cancer cell lines may be a consequence of the manner in which the CXCL12–KRT19 coating is formed. KRT19, which is expressed by all epithelial cancers (18), is the central player in that it binds both CXCL12 and TGM2. KRT19 binds TGM2 only in buffer containing Ca^2+^, suggesting that the interaction occurs only with the enzyme in its “open”, enzymatically active conformation. This finding with recombinant proteins replicates an earlier observation that TGM2 and KRT19 form a complex in the cytosol of the SKBR3 breast cancer line, which, however, is a site that would favor the closed conformation of TGM2 because of its high guanosine-5′-triphosphate (GTP) and low Ca^2+^ concentrations (11, 19). The two observations may be reconciled by the additional finding that the intracellular binding of KRT19 by TGM2 required its active site cysteine, which would be exposed to TGM2 in its open conformation. This intracellular complex may represent the thioester-linked TGM2–KRT19 complex that, upon secretion, would be subject to nucleophilic attack by CXCL12 after binding to KRT19, thereby forming the isopeptide bond linking the two proteins. The specificity of the CXCL12–KRT19 heterodimer, therefore, would be explained by the combination of the intracellular TGM2–KRT19 interaction with the extracellular KRT19–CXCL12 interaction. Intracellular formation of the thioester-linked complex between TGM2 and KRT19 would be consistent with the requirement that PDA cells express the enzyme (Fig. 5).

That CXCL12 is cross-linked to KRT19 allows the heterodimer to self-assemble with KRT8, which is also secreted (20), into a higher-order filamentous network that coats the cancer cell. This organization of the CXCL12–KRT19 heterodimers on cancer cells would be capable of cross-linking CXCR4 on adjacent T cells, prompting our studies with the (CXCL12)_2_–Fc fusion protein. Directly visualizing movement of the human T lymphoblastoid cells, rather than performing a two-chamber chemotaxis assay, revealed that the (CXCL12)_2_–Fc protein induced a CXCR4 “stop” signal that suppressed the spontaneous motility of the T cells. This finding, which may be related to the inhibition of F-actin polymerization by a different form of CXCL12 dimer (15), provides insight into the exclusion of T cells by the CXCL12–KRT19 coating of cancer cells. It also may explain the puzzling report that an anti-CXCR4 antibody, which blocks CXCL12 binding, does not release hematopoietic stem cells (HSCs) from their bone marrow niches (21). This anti-CXCR4 IgG, like the (CXCL12)_2_–Fc fusion protein, may cross-link CXCR4 to stimulate a CXCR4 “stop” signal that prevents the egress of HSCs. The unbound CXCL12 in the TME of PDA tumors lacking the CXCL12–KRT19 coating will elicit the other immune-suppressive effect of CXCR4, which is inhibition of other chemokine receptors to mediate directed migration (8). This circumstance may explain the more marked effect on PDA growth of blocking all CXCR4 inhibitory functions with the CXCR4 inhibitor AMD3100 (7) than of preventing only the formation of the CXCL12–KRT19 coating.

Finally, our experiments demonstrate that among the factors that contribute to an absence of intratumoral T cells, the capacity of cancer cells to form the CXCL12–KRT19 coating is dominant, and the exclusion of T cells by this means alone results in resistance to inhibition of the PD-1 checkpoint. Since at least three human carcinomas exhibit the CXCL12–KRT19 coating, all of which typically do not respond to immunotherapy with anti–PD-1 antibody, suppressing the formation of the coating may be therapeutically beneficial. Although normal epithelial cells may also form the CXCL12–KRT19 coating (22), an absence of its formation may be tolerated since mice with knockout of *Krt19* or *Tgm2* exhibit no spontaneous abnormalities (23, 24).

## Materials and Methods

### Human Tumor Samples.

All human tumor samples used in this study are approved by the institutions’ Institutional Review Board. Freshly resected human pancreas and colon tumors were obtained from Northwell Health Tissue Donation Program. The collected tumor samples were temporarily stored in Gibco Roswell Park Memorial Institute 1640 (RPMI-1640) medium and delivered on ice the same day of surgical removal. After receiving, part of each sample was embedded in Tissue-Tek optimal cutting temperature (OCT) compound (Sakura, 4583) for immunofluorescence staining, and the rest was flash-frozen in liquid nitrogen for protein extraction. Formalin-fixed paraffin-embedded (FFPE) human MSS and MSIhigh pancreatic tumor slices were obtained from Memorial Sloan Kettering Cancer Center.

### Tumor Protein Extraction.

Tumor pieces of about 100 mg were used for protein extraction. Freshly dissected tumor pieces or the tumor pieces that were previously flash-frozen in liquid nitrogen and subsequently stored at −80 °C were briefly homogenized in 1 mL of ice-cold IP buffer (75 mM HEPES, pH 7.5, 150 mM NaCl, 1 mM dithiothreitol (DTT), 10% glycerol, and protease inhibitor mixture; Thermo, 78437) plus 1% Nonidet P-40. After removal of remaining large tissue particles by flowing through the Pierce Tissue Strainer (Thermo, 87791), the homogenized tissue was sequentially extracted using the following stepwise instructions. First, resuspend the tissue and incubate at 4 °C on a rotator for 10 min followed by centrifugation at 4,000 × *g* for 5 min. Save the supernatant as “NP-40 extract”. Repeat to wash the pellet. Second, resuspend the pellet in 250 μL of IP buffer plus 0.1% sodium DOC and incubate at 4 °C on a rotator for 30 min, followed by centrifugation at 6,000 × *g* for 5 min. Save the supernatant as “DOC extract”. Repeat to wash the pellet. Third, resuspend the pellet in 200 μL of IP buffer plus 5 mM CaCl_2_ and 3,000 U/mL micrococcal nuclease (Thermo, 88216) and incubate at room temperature for 30 min followed by centrifugation at 16,000 × *g* for 5 min. Save the supernatant as “DNase extract”. Repeat to wash the pellet. Fourth, resuspend the pellet in 150 μL of IP buffer plus 1% SDS and incubate at room temperature for 10 min, followed by centrifugation at 16,000 × *g* for 5 min. Keep the supernatant as “SDS extract”, which is the fraction of cytoskeleton. For the extractions with TGM2 inhibition, 100 μg/mL or 500 μg/mL ERW1041E (Sigma, 509522) or an equivalent volume of dimethyl sulfoxide (DMSO) was included in every extraction buffer. Protein concentration was measured using a protein assay kit (Bio-Rad, 5000112), and 1 μg of each fraction was boiled in Laemmli sample buffer containing 20 mM DTT and applied to an SDS-PAGE gel for Western blotting.

### IP of the CXCL12–KRT19 Coating.

SDS in the tumor cytoskeleton fraction was removed with the Pierce Detergent Removal Spin Column (Thermo, 87777). For IP, 100 μg of the protein extract was diluted in 500 μL of IP buffer containing 1% bovine serum albumin (BSA; Calbiochem, 2930) and 0.1% SDS, which helps to prevent self-assembly of cytokeratin proteins. The mixture was precleared by centrifugation at 16,000 × *g* for 10 min, and the supernatant was transferred into a new tube. Then, 5 μg of rabbit isotype IgG (Thermo, 10500C) or rabbit anti-CXCL12 antibody (LSBio, LS-C48888) was added to the solution and incubated on a rotor at 4 °C overnight. Separately, 100 μL of protein G agarose beads (Cell Signaling, 37478; 50% slurry) were preblocked in IP buffer containing 1% BSA. After overnight incubation, 20 μL of pure protein G resins (or 40 μL of 50% slurry) was added directly to the protein/antibody solution and incubated for another 2 h at 4 °C. Following three washes with IP buffer containing 0.1% SDS, the resins were boiled in 100 μL of Laemmli sample buffer containing 20 mM DTT, and 10 μL of each sample was applied to an SDS-PAGE gel for Western blotting, where Veriblot-HRP (Abcam, ab131366) was used.

### Cell Surface Protein Biotinylation and Isolation.

SKBR3 and Caco2 cells were cultured to 95% confluency in 10-cm plates. The cells were washed once with phosphate-buffered saline (PBS), and then 4 mL of 0.5 mg/mL Sulfo-NHS-LC-Biotin (Thermo, A39257) in PBS was added to the dish for 10 min at room temperature. After, the plates were washed twice with 10 mL of cold Tris-buffered saline (Thermo, 28376), and then the cells were harvested by scraping in 10 mL of the cold Tris-buffered saline with protease/phosphatase inhibitor mixture (Thermo, 78445). The cells were pelleted and lysed with 800 μL of cold SDS-containing radio-immunoprecipitation assay (RIPA) buffer with protease/phosphatase inhibitor. The protein concentration was quantified by bicinchoninic acid (BCA; Thermo, 23225); 250 μg of the lysate was incubated with 250 μL of a 50% NeutrAvidin agarose (Thermo, 29200) slurry in 300 μL of RIPA buffer with protease/phosphatase inhibitor for 30 min. The beads were them washed four times with RIPA buffer with protease/phosphatase inhibitor, and the beads were eluted by boiling in Laemmli sample buffer containing 20 mM DTT.

### Cell Line IP.

SKBR3 and Caco2 cells were cultured to 95% confluency in 10-cm plates. The cells were washed once with PBS and harvested by scraping in 10 mL of cold Tris-buffered saline (Thermo, 28376) with protease/phosphatase inhibitor mixture (Thermo, 1861284). The cells were pelleted and resuspended in 700 μL of cold 1% Triton X-100–containing lysis buffer (25 mM Tris pH 7.4, 100 mM NaCl, 1 mM EDTA) with the protease/phosphatase inhibitor. The protein concentration was quantified by BCA. Four hundred milligrams of lysate was added to 5 mg of anti-KRT19 (Abcam, ab76539) antibody or IgG control (Cell Signaling, 3900S) and incubated overnight at 4 °C. Thirty-five microliters of a 50% Protein G Dynabead (Thermo, 10004D) slurry was blocked overnight in 1% BSA at 4 °C for each antibody. After the overnight incubation, the beads were added to the antibody–antigen solution for 2 h at 4 °C, after which the beads were washed three times with lysis buffer before boiling in 100 μL of Laemmli sample buffer containing 20 mM DTT. Sample (5 to 15 μL) was applied to an SDS-PAGE gel for Western blotting, where Veriblot-HRP (Abcam, ab131366) was used.

### Fetal Calf Serum IP.

Fifteen micrograms of biotinylated anti-CXCL12 (Peprotech, 500-P87ABT) or IgG control antibody (Abcam, ab200208) was preabsorbed onto 3 mg of Dynabeads MyOne Streptavidin T1 (Thermo, 65601). This antibody–bead complex was then incubated with 10 mL of fetal calf serum (Seradigm, 1500-500) overnight. The antibody–bead complex was removed and washed with PBS before being boiled in Laemmli sample buffer containing 20 mM DTT. The sample was then applied to an SDS-PAGE gel for Western blotting.

### Protein-Binding Assays and Cross-Linking.

The buffer of commercially available recombinant human KRT19 protein (Creative Biomart, KRT19-7239H) was changed to protein-binding buffer (25 mM Tris, pH 7.5, 100 mM NaCl, 1% Triton X-100) using Zeba spin desalting columns (Thermo, 89882). For protein binding, biotinylated recombinant human CXCL12 (Chemotactics, B-CXCL12) or CXCL8 (Chemotactics, B-CXCL8) was incubated with KRT19 at 4 °C for 2 h. Then, streptavidin-conjugated Dynabeads (Thermo, 11205D), preblocked with 1% BSA, were added and incubated for another 2 h at 4 °C. Following three washes, bound proteins were boiled in Laemmli sample buffer containing 20 mM DTT for Western blotting. For saturation binding between CXCL12 and KRT19, 100 ng of CXCL12–biotin bound on streptavidin Dynabeads was incubated with 0 nM, 8 nM, 16 nM, 31 nM, 63 nM, 125 nM, or 250 nM KRT19 at 4 °C for 4 h. For TGM2 binding, CXCL12–biotin or KRT19/CXCL12–biotin complex was immobilized on streptavidin Dynabeads. Then, recombinant human TGM2 (R&D Systems, 4376-TG-050) was added and incubated in the presence or absence of 10 mM CaCl_2_ on ice for 10 min. Following two quick washes on ice with calcium-containing binding buffer, bound proteins were boiled for Western blotting. For KRT19–CXCL12 cross-linking, 100 ng of CXCL12–biotin and 500 ng of KRT19 were incubated and bound on streptavidin Dynabeads. After three washes, KRT19/CXCL12–biotin complex-bound Dynabeads were resuspended in 50 μL of TGM2 activation buffer (100 mM Tris, pH 7.5, 150 mM NaCl, 10 mM CaCl_2,_ 10 mM DTT). Then, 50 ng of recombinant human TGM2, together with or without 100 μM ERW1041E, was added and incubated at room temperature for 15 min. The reactions were stopped by direct boiling in Laemmli sample buffer containing 20 mM DTT.

### Animals.

C57BL/6 mice of 6 wk of age were purchased from The Jackson Laboratory. After receiving, the mice were housed in a *Helicobacter*-free room for 2 wk before each experiment. TGM2-KO mice were obtained by cross-breeding *Tgm2*-floxed mice (Jackson, 024694) to transgenic CMV-Cre mice (Jackson, 006054). The progeny were genotyped at Transnetyx for double-allele KOs. All animal experiments were approved by the Cold Spring Harbor Laboratory (CSHL) Institutional Animal Care and Use Committee in accordance with the NIH Guide for the Care and Use of Laboratory Animals.

### CRISPR Editing and Lentivirus.

The control CRISPR guide [sgScramble: GCTTAGTTACGCGTGGACGA (25)] and the guides targeted to the mouse *Krt19* gene (sgKRT19 [1]: CACAGGAAATTACTGCCCTG; sgKRT19 [2]: TAGTGGTTGTAATCTCGGGA; sgKRT19 [3]: CGGAGGACGAGGTCACGAAG) or mouse *Tgm2* gene (sgTGM2 [1]: GAATATGTCCTTACGCAACA; sgTGM2 [2]: GTCCTGTTGGTCCAGCACTG; sgTGM2 [3]: TTGACCTCGGCAAACACGAA) were cloned into the lentiCRISPR-v2 plasmid (Addgene, 52961) and sequencing validated. The lentivirus was produced by cotransfecting HEK293T cells with the guide containing lentiCRISPR-v2 plasmid together with the lentivirus-packing plasmid psPAX2 (Addgene, 12260) and the envelope-expressing plasmid pMD2.G (Addgene, 12259).

### Cancer Cell Lines.

The mouse PDA cancer cell line 1242, derived from LSL-Kras^G12D/+^; LSL-Trp53^R172H/+^; Pdx1-Cre (KPC) mouse tumor (26), was a gift from the laboratory of Dr. Tuveson. To generate sgScramble, sgKRT19, and sgTGM2 cell lines, 1,242 cells were transduced with lentivirus expressing SpCas9 and specific CRISPR guides. The transduced cells were directly selected with 1 μg/mL puromycin (Sigma, P8833) for polyclonal stable cell lines. The single-cell clones were generated by limited dilution. To rescue KRT19 or TGM2 expression in the *Krt19*-edited or *Tgm2*-edited cancer cells, which was achieved by transduction with the lentivirus containing the sgKRT19(1) guide or the sgTGM2(1) guide, the single-cell clone was further transduced with lentivirus expressing mouse KRT19 with four synonymous mutations or mouse TGM2 with six synonymous mutations at the guide targeting site. To facilitate tumor imaging in vivo for hepatic metastasis, the sgScramble, sgKRT19, and sgTGM2 cell lines were transduced with lentiviral plasmid expressing firefly luciferase and green fluorescent protein (GFP), which are linked by 2A peptide. The luciferase/GFP-transduced cells were sorted by fluorescence-activated cell sorting (FACS). Dox-inducible ovalbumin-expressing cells were generated by transducing 1,242-sgScramble cells and 1,242-sgKRT19 cells with the lentiviral vector pCW57-gfp-p2a-mcs (Addgene, 89181) containing the cytoplasmic version of ovalbumin followed by FACS and single-cell cloning. All the mouse cancer cell lines were cultured in Dulbecco’s modified Eagle medium (DMEM; Corning, 10-013-CV) supplemented with 10% fetal bovine serum (FBS; Seradigm, 1500-500), 100 U/mL penicillin, and 100 μg/mL streptomycin. Human SKBR3 and Caco2 cell lines were procured from the CSHL cell line repository and cultured in DMEM (Corning, 10-013-CV) supplemented with 10% FBS (Seradigm, 1500-500), 100 U/mL penicillin, and 100 μg/mL streptomycin. For serum-free experiments, SKBR3 cells were cultured in Cancer Cell Line Medium XF (PromoCell, C-28077), a xeno-free complete medium. Prior to culture, dishes were briefly coated with 2 μg/cm^2^ human plasma fibronectin (Sigma, FC010), while glass-bottomed tissue culture plates (Chemglass, CLS-1812-024) were coated overnight.

### Mouse Studies.

For subcutaneous tumors, 2.5 × 10^5^ cells, unless indicated otherwise, were injected into the right flank of mice. Tumor volume was measured with a caliper every 2 or 3 d and calculated according to the formula *V* = (*L* × *W*^2^)/2. Mice that were developing a tumor-associated skin ulcer during the experiment were humanely killed, and visually ulcerated tumors were not included in further analysis. For the hepatic metastasis model, 2.5 × 10^4^ to 1.0 × 10^5^ luciferase-expressing cells were injected into the portal vein. Tumor size was monitored through bioluminescence imaging every 3 or 4 d. To do so, 150 μL of 30 mg/mL D-Luciferin (PerkinElmer, 122799-10) was administered through intraperitoneal injection, and mice were imaged 10 min after D-Luciferin administration with the IVIS Spectrum In Vivo Imaging System. For mouse tumor immunotherapy, 200 μg of isotype IgG (BioXcell, BP0089) or rat anti–mouse PD-1 antibody (BioXcell, BP0273) was given through intraperitoneal injection on the indicated days. All mice that were developing tumor-associated ascites or lethargy during the experiment were humanely killed. For the ovalbumin-inducible 1242-sgScramble and 1242-sgKRT19 s/c tumors, 5 × 10^5^ cells were inoculated, and ovalbumin was induced using Dox food at day 0.

### Immunofluorescence.

Mouse tumors were embedded in Tissue-Tek OCT compound (Sakura, 4583) and freshly frozen on dry ice. The embedded tumors were sectioned at 10 μm for staining. Before staining, the sections were fixed with freshly prepared fixation buffer (PBS plus 4% paraformaldehyde) for 15 min at room temperature followed by three washes with PBS. For human FFPE tumor sections, the slides were deparaffinized in xylene three times (5 min each) followed by sequential rehydration treatments in 100% ethanol (5 min, three times), 95% ethanol (5 min), 70% ethanol (5 min), and water (5 min). After rehydration, antigen retrieval was conducted by boiling the sample in 10 mM citrate, pH 6.0, plus 0.05% Tween 20 for 10 min, followed by cooling down to room temperature for 30 min and then two washes in water for 5 min each. To stain tissue sections, a hydrophobic barrier was drawn around the tumor slice with an ImmEdge PAP pen (Vector Laboratories, H-4000). The sections were then permeabilized with 0.2% Triton X-100 in PBS for 15 min followed by blocking with 1% BSA (Calbiochem, 2930) or 10% normal goat serum (Thermo, 16210064) in PBS at room temperature for 1 h. After blocking, the tumor slices were stained by incubation with nonconjugated primary antibodies (*SI Appendix*, Table S1) at 4 °C overnight and subsequently with fluorophore-conjugated secondary antibodies at room temperature for 1 h. When fluorophore-conjugated primary antibodies (*SI Appendix*, Table S1) were used, the sections were incubated with the antibodies at room temperature for 2 h. Nuclei were stained with DAPI (Thermo, R37606) at room temperature for 10 min. In between each step, sections were washed twice with 0.05% Tween 20 in PBS and once with PBS for 5 min for each wash. After the stainings, tumor sections were mounted with mounting medium (Thermo, P36961). For the staining of the thick (30-μm) nonpermeabilized section, Triton X-100 was not included. Images were acquired on a Leica SP8 confocal microscope or ZEISS Observer microscope and analyzed with ImageJ software.

### Live-Cell Staining.

SKBR3 and Caco2 cells were cultured to 95% confluency in glass-bottomed tissue culture plates (Chemglass, CLS-1812-024). Prior to staining, cells were washed once with PBS, and then in cold 0.1% BSA, 250 μL of 1 mg/mL antibody mixture was added to each well. The antibody and cells were incubated together at 4 °C for 30 min, protected from light, after which the cells were washed three times with cold PBS. The cells were then fixed with 4% paraformaldehyde and stained with 20 mM Hoechst 33342 (Thermo, 62249).

### Flow Cytometry.

Freshly resected tumors were minced and digested in complete DMEM cell culture medium containing 250 μg/mL collagenase D (Sigma, 11088858001), 50 μg/mL Liberase DL (Sigma, 05466202001), and 20 μg/mL DNase I (Sigma, 10104159001) at 37 °C for 45 min with shaking. Following lysis of red blood cells (RBCs) in RBC lysis buffer (155 mM NH_4_Cl, 12 mM NaHCO_3_, and 0.1 mM EDTA), cells were passed through a 70-μm cell strainer and resuspended in DMEM containing 50 μg/mL DNase I to digest released DNA from RBCs. After incubation at room temperature for 15 min, the cells were washed and resuspended in FACS buffer (PBS, 2% FBS, and 20 mM HEPES, pH 7.4). For staining, the cells were first blocked with rat anti-mouse CD16/CD32 antibody (BioLegend, 101302) in FACS buffer with shaking at 4 °C for 30 min. Then, fluorophore-conjugated antibodies (*SI Appendix*, Table S1) were added and incubated for another 30 min in the dark, followed by two washes with FACS buffer. Calcein violet 450 AM (Thermo, 65-0854-39) or DAPI (Thermo, R37606) was used for live-cell gating. Data were acquired using a BD LSRFortessa cell analyzer equipped with Diva software 6.0 and were further analyzed with FlowJo.

### qPCR.

Tumor samples of about 30 mg were flash-frozen in liquid nitrogen and stored at −80 °C. Total RNA was extracted with an RNeasy mini kit (Qiagen, 74106) according to the manual. To make the cDNA library, 1 μg of total RNA was reverse transcribed with a TaqMan Reverse Transcription kit (Thermo, N8080234) using oligo d(T)s. The cDNA library was then diluted in 100 μL of nuclease-free water. For qPCR, 5 μL of diluted cDNA library was mixed in a 384-well plate with specific TaqMan probes (*SI Appendix*, Table S2) and TaqMan Universal Master Mix II (Thermo, 4440040) to make 10-μL reaction volumes. The qPCRs were run in QuantStudio 6. All the gene expression values were normalized to *Tbp*.

### RT-PCR.

SKBR3 and Caco2 cell line RNA was extracted according to the instructions of the RNeasy mini kit (Qiagen, 74106). To make the cDNA library, 1 μg of total RNA was reverse transcribed with a TaqMan Reverse Transcription kit (Thermo, N8080234) using oligo d(T)s. For RT-PCR, 5 μL of undiluted cDNA library was mixed in a 384-well plate with specific TaqMan probes (*SI Appendix*, Table S2) and TaqMan Universal Master Mix II (Thermo, 4440040) to make 10-μL reaction volumes. Replicate reactions were pooled after amplification and run on a 2% agarose gel in Tris-borate-EDTA buffer.

### RNA-seq.

Subcutaneous tumors were harvested 2 wk after cancer cell inoculation. The sequencing libraries were prepared according to the Illumina TruSeq Stranded Total RNA Sample Preparation Guide, with certain modifications. Briefly, 4 μg of total RNA of each sample was used to make the cDNA library. To enrich DNA fragments at the final step, 12 PCR cycles, instead of 15, were adopted. For library pooling, each cDNA library was diluted to 10 nM, and 30 μL of each sample was pooled. The final pooled library was submitted to the CSHL Sequencing Core for high-throughput next-generation sequencing, with the sequencing type of single-read 76 base pairs (bp) plus bar code.

### Cell Motility Assays.

To express and purify dimeric (CXCL12)_2_–Fc, human CXCL12α cDNA devoid of the intrinsic signal peptide sequences was cloned into pFUSE-hIgG4-Fc2 (Invitrogen, pfuse-hg40fc2 containing human interleukin-2 [IL2] signal sequence) plasmid using EcoRI and XhoI restriction sites. HEK293T cells transfected with the plasmid were cultured in DMEM (Corning, 10-013-CV) containing 10% IgG-low FBS (Corning, 35-073-CV) for 48 h, and the cultured medium was collected for protein purification using protein G columns (Thermo, 89957). Human CCRF-HSB-2 T lymphoblast cells (ATCC, CCL-120.1) were maintained at a density of 1 × 10^5^ to 1 × 10^6^ cells/mL in RPMI-1640 medium (Corning, 10-040-CV) supplemented with 10% FBS (Seradigm, 1500-500), 100 U/mL penicillin, and 100 μg/mL streptomycin. One day before each experiment, cells were counted and changed to new medium to continue culturing. The next day, cells were collected and washed twice with chemotaxis buffer (RPMI-1640 medium plus additional 20 mM HEPES, pH 7.5, and 0.5% BSA) and resuspended in chemotaxis buffer at a density of 1 × 10^6^ cells/mL. Then, 50 μL of cell suspension and 50 μL of 2 mg/mL collagen (Platypus, CI48) were mixed together with the indicated concentration of (CXCL12)_2_–Fc protein followed by flowing in 30 μL to each individual imaging channel of the Ibidi μ slide VI-Flat chamber (Ibidi, 80626). After a 30-min incubation at 37 °C to allow collagen gelation, cells were imaged with a ZEISS Observer microscope every 3 min under 5% CO_2_ for 2 h. For data analysis, cell movements were manually tracked with ImageJ software and analyzed with the Ibidi Chemotaxis and Migration Tool (https://ibidi.com/chemotaxis-analysis/171-chemotaxis-and-migration-tool.html).

## Supplementary Material

Supplementary File

## Data Availability

RNAseq data for Fig. 4*F* have been deposited in the Gene Expression Omnibus database (accession nos. GSE192853 and GSE193027).All other study data are included in the manuscript and/or *SI Appendix*.
