# Supplementary material for: Carcinomas assemble a filamentous CXCL12–keratin-19 coating that suppresses T cell–mediated immune attack
Source: Proc Natl Acad Sci U S A. 2022 Jan 19;119(4):e2119463119. doi: 10.1073/pnas.2119463119 (PMC8794816; doi:10.1073/pnas.2119463119)
Supplement: Supplementary File [file pnas.2119463119.sapp.pdf]

Supplementary Information for

Carcinomas assemble a filamentous CXCL12-keratin19 coating that suppresses T cell-mediated immune attack

Zhikai Wang[a], Philip Moresco[a,b,c, 1], Ran Yan[a,d, 1], Jiayun Li[a], Ya Gao[a], Daniele Biasci[e], Min Yao[a], Jordan Pearson[a,b,c], Jaclyn F. Hechtman[f], Tobias Janowitz[a,g], Raza M. Zaidi[g,h], Matthew J. Weiss[g], and Douglas T. Fearon[a,i,*]

[*]To whom correspondence should be addressed: Douglas T. Fearon. Email: dfearon@cshl.edu

This PDF file includes:

Figures S1 to S15
Tables S1 to S2

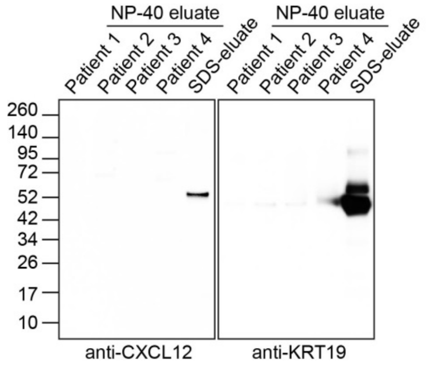

Figure S1: Characterization of the CXCL12-KRT19 coating of human adenocarcinomas. The NP-40 eluates of the human PDA and CRC tumors, shown in Fig. 1B, were subjected to SDS-PAGE and immunoblotting with anti-CXCL12 or anti-KRT19 antibodies. The SDS eluate of patient 2 was applied as a positive control.

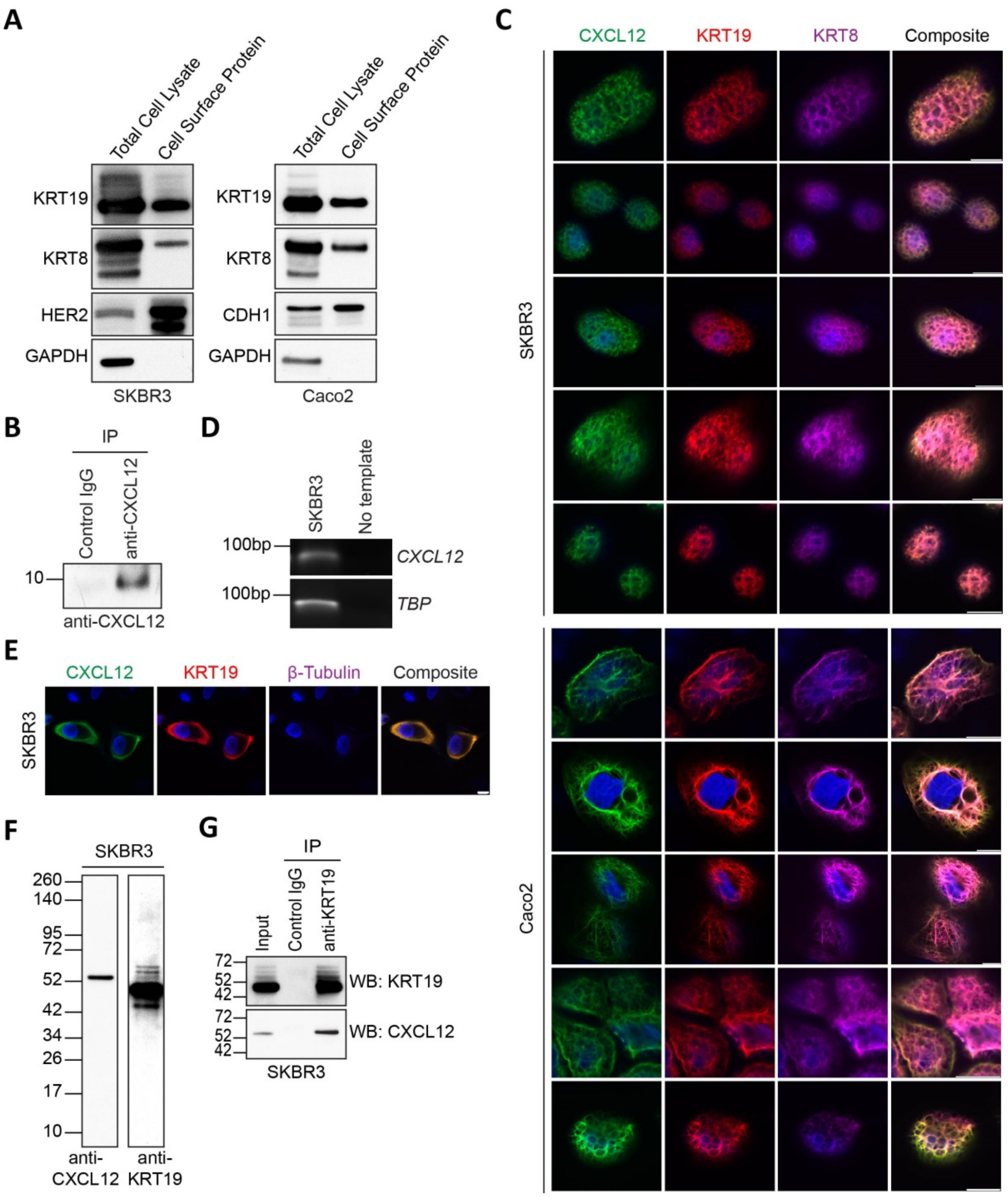

Figure S2: The formation of keratin intermediate filaments on the surface of live intact cancer cell lines. (**A**) Human breast cancer cells and colon cancer cells, SKBR3 and Caco2, respectively, were subjected to surface protein biotinylation followed by purification with SA-coated microbeads. Total cell lysates and purified surface biotinylated protein were applied to SDS-PAGE and immunoblotted with antibodies to KRT19, KRT8, HER2, CDH1 and GAPDH. (**B**) The proteins that were immunoprecipitated by isotype control IgG or anti-CXCL12 antibody from fetal calf serum were subjected to SDS-PAGE and immunoblotting with anti-CXCL12 antibody. (**C**) Cultured SKBR3 and Caco2 cells were stained with fluorochrome-conjugated antibodies to KRT19 and CXCL12 prior to fixation and permeabilization. Five representative confocal images are shown. Scale bars, 10□m. (**D**) mRNA was extracted from SKBR3 cells and *CXCL12* and *TBP* cDNA fragments were PCR amplified and subjected to agarose gel electrophoresis. (**E**) SKBR3 cells that were cultured in serum free medium were stained with fluorochrome-conjugated antibodies to CXCL12, KRT19 and β-tubulin prior to fixation and permeabilization. (**F**) SDS-eluates of SKBR3 cells that were cultured in serum-free medium were subjected to SDS-PAGE

and immunoblotting with anti-CXCL12 or anti-KRT19 antibody. (**G**) The proteins that were immunoprecipitated by isotype control IgG or anti-KRT19 antibody from SKBR3 cells cultured in serum-free medium were subjected to SDS-PAGE and immunoblotting with anti-KRT19 and anti-CXCL12 antibodies.

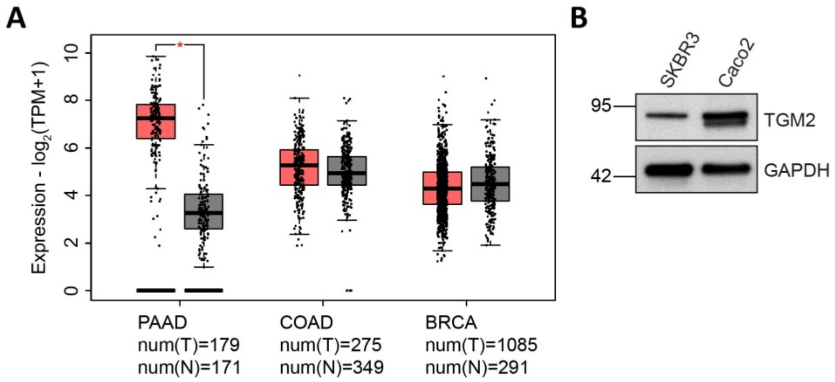

Figure S3: Expression of TGM2 in human PDA, CRC and breast tumors and cancer cell lines. (**A**) Box plots showing TGM2 expressions in PDA (PAAD), CRC (COAD) and breast tumors (BRCA) (red), and corresponding normal tissues (gray). Data were derived from http://gepia.cancer-pku.cn/index.html. (**B**) Total protein extracts from SKBR3 and Caco2 cancer cells were subjected to SDS-PAGE and immunoblotting with anti-TGM2 and anti-GAPDH antibodies.

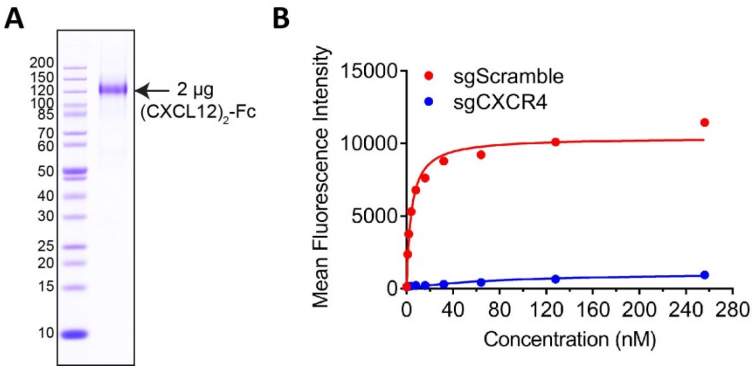

Figure S4: Purified $(CXCL12)_2$-Fc and its binding to sgScramble or sgCXCR4 edited human T lymphoblastoid cells. (**A**) 2 µg $(CXCL12)_2$-Fc protein purified from HEK293T cells was subjected to non-reducing SDS-PAGE and staining with Coomassie Brilliant Blue dye. (**B**) sgScramble or sgCXCR4 edited Jurkat cells (*8*) were incubated with increasing concentrations of $(CXCL12)_2$-Fc. After a brief wash, cells were stained with fluorochrome-conjugated goat anti-human IgG and subjected to flow cytometry analysis. Mean fluorescence intensities were plotted against $(CXCL12)_2$-Fc concentrations.

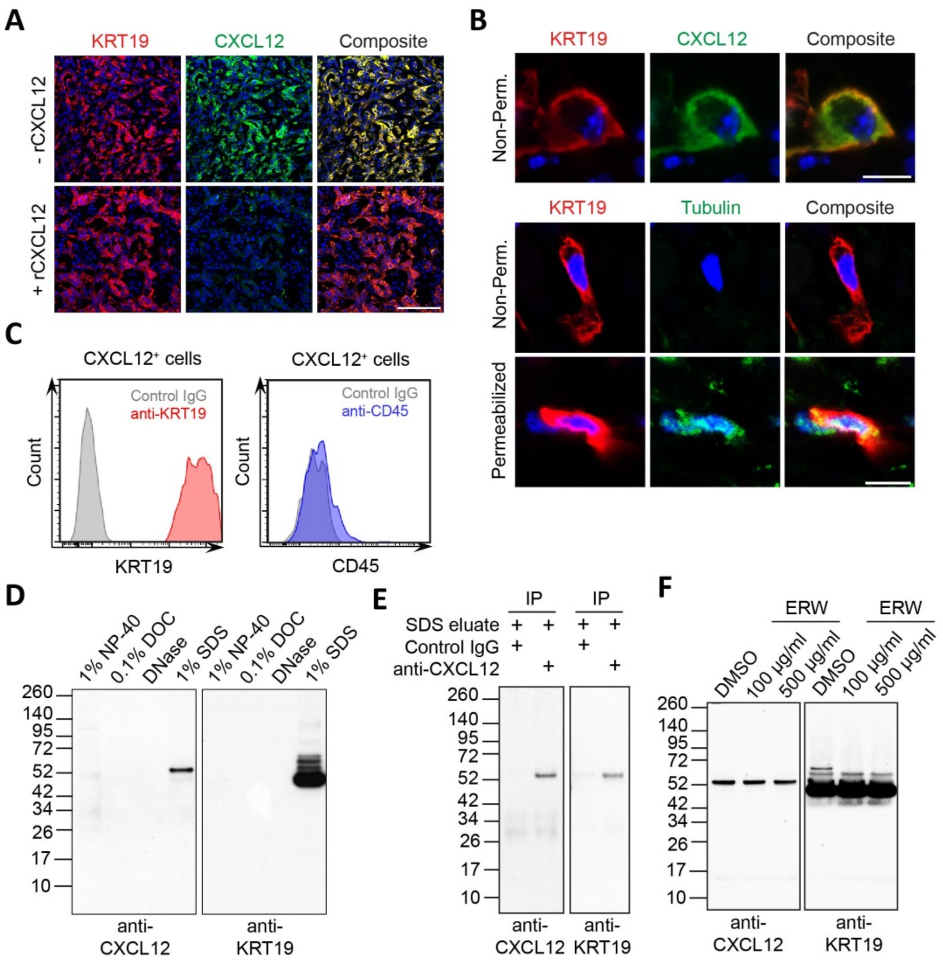

Figure S5: Characterization of the CXCL12-KRT19 coating of mouse PDA tumors. (**A**) Frozen sections of a mouse PDA tumor were stained with fluorescent antibodies to KRT19 and CXCL12, in the absence or presence of 1 µg/mL recombinant CXCL12 protein (rCXCL12). Scale bar, 100 µm. (**B**) 30 µm-thick sections of a mouse s/c PDA were permeabilized with Triton X-100, or not, were stained with fluorescent antibodies to KRT19, CXCL12, and tubulin. Scale bars, 10 µm. (**C**) Intact, DAPI-excluding, dissociated cells from a mouse s/c PDA tumor that had been stained with fluorescent antibodies to CXCL12, KRT19 and CD45, were assessed by flow cytometry. (**D**) Sequential detergent eluates of a mouse s/c PDA were subjected to SDS-PAGE and immunoblotting with anti-KRT19 or anti-CXCL12 antibody. (**E**) The proteins that were immunoprecipitated by control IgG or anti-CXCL12 antibody from the SDS-eluate of a mouse s/c PDA were subjected to SDS-PAGE and immunoblotting with anti-CXCL12 or anti-KRT19 antibodies. (**F**) Sequential extractions of mouse s/c PDA were performed in the presence of 100 µg/ml, 500 µg/ml ERW1041E, or DMSO, and the SDS eluates were analyzed by SDS-PAGE and immunoblotting with anti-CXCL12 or anti-KRT19 antibodies.

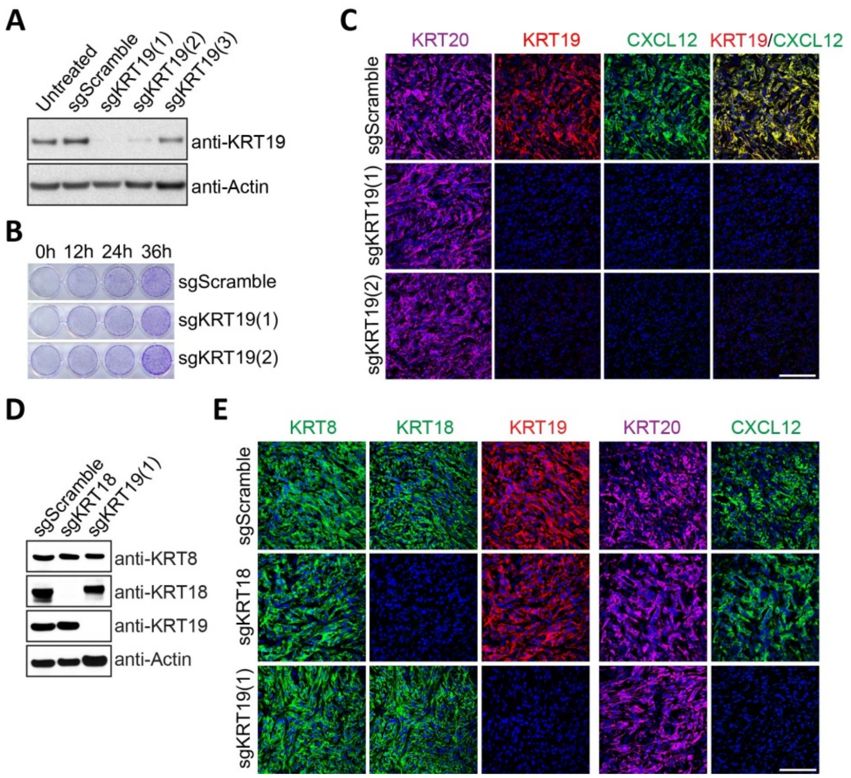

Figure S6: *Krt19*-edited PDA cells and the formation of the CXCL12-KRT19 coating. (**A**) Lysates from mouse PDA cell lines that were CRISPR/Cas9 edited with scramble sgRNA or three different *Krt19* sgRNAs were subjected to SDS-PAGE and immunoblotting with anti-KRT19 or anti-actin antibodies. (**B**) Crystal violet stains measuring the *in vitro* growth of sgScramble and sgKRT19-edited PDA cells are shown. (**C**) Tissue sections of s/c tumors formed by control and the indicated two *Krt19*-edited PDA cell lines were stained with fluorescent antibodies to KRT20 to reveal cancer cells, KRT19 and CXCL12, respectively. (**D**) Lysates from mouse PDA cell lines that were CRISPR/Cas9 edited with scramble sgRNA, sgKRT18 or sgKRT19 were subjected to SDS-PAGE and immunoblotting with anti-KRT8, anti-KRT18, anti-KRT19 or anti-actin antibodies. (**E**) Sections of s/c tumors formed with sgScramble-, sgKRT18- or sgKRT19-edited PDA cells were stained with fluorescent antibodies to KRT8, KRT18, KRT19, KRT20 and CXCL12, respectively. Scale bars, 100 μm.

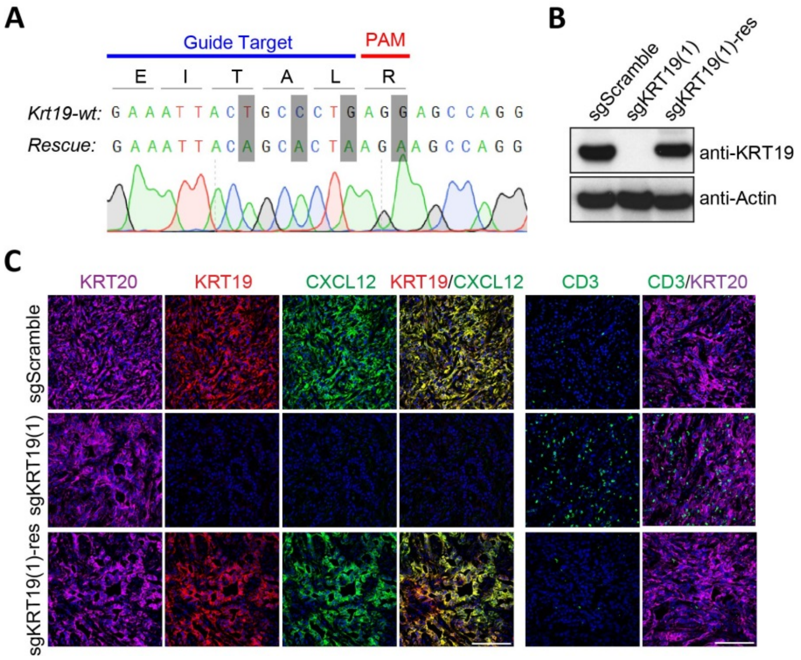

Figure S7: Rescue of KRT19 expression in *Krt19*-edited PDA cells. (**A**) A KRT19 "rescue" construct in which four synonymous mutations, shown in black boxes, were introduced to prevent the binding of sgKRT19(1). (**B**) PDA cell lysates were subjected to SDS-PAGE and immunoblotting with anti-KRT19 and anti-actin antibodies. (**C**) Tissue sections of s/c tumors formed with the sgScramble PDA cells, sgKRT19-edited PDA cells, and the KRT19-rescue PDA cells were stained with fluorescent antibodies to KRT20 to reveal cancer cells, KRT19, CXCL12, and CD3, respectively. Scale bars, 100 μm.

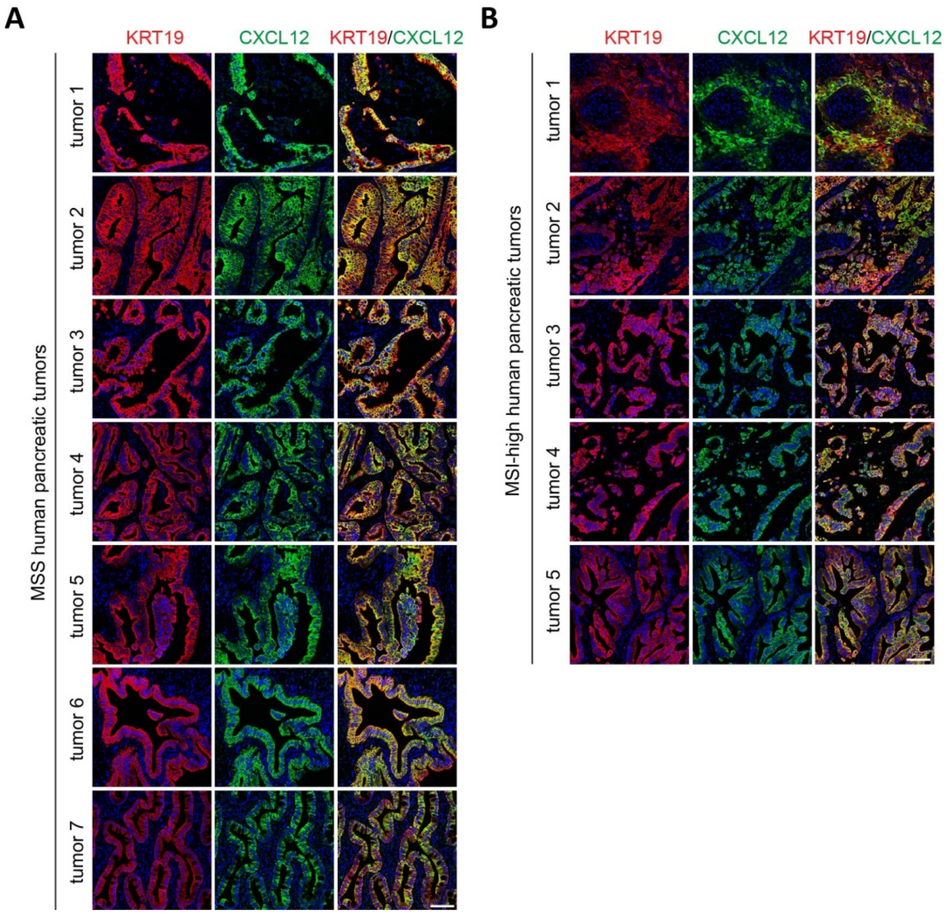

Figure S8: The CXCL12-KRT19 coating of cancer cells in MSS and MSI-high PDA. (**A-B**) Sections of FFPE human MSS (A) and MSI-high (B) PDA were stained with fluorescent antibodies to KRT19 and CXCL12.

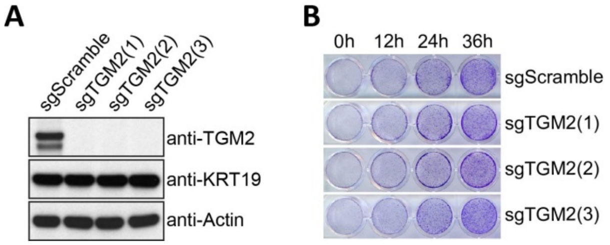

Figure S9: *Tgm2*-edited PDA cells. (**A**) Lysates from mouse PDA cell lines that were CRISPR/Cas9 edited with scramble sgRNA or three different *Tgm2* sgRNAs were subjected to SDS-PAGE and immunoblotting with anti-TGM2, anti-KRT19 or anti-actin antibodies. (**B**) *In vitro* growth of sgScramble and sgTGM2-edited PDA cells was assessed by staining every 12 hours with crystal violet.

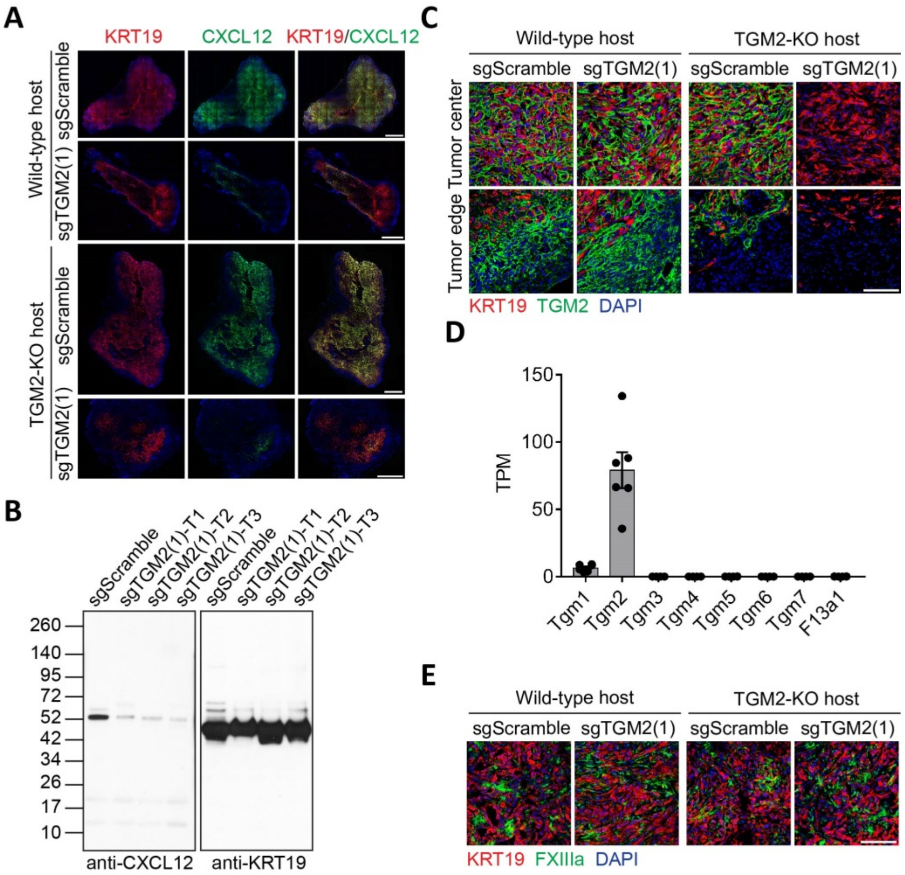

Figure S10: The role of TGM2 expression by mouse PDA cells in the formation of the CXCL12-KRT19 coating. (**A**) Sections of s/c tumors formed with sgScramble or sgTGM2-edited PDA cells, in wild-type hosts or TGM2-KO hosts, were stained with fluorochrome-conjugated antibodies to KRT19, CXCL12, and CD3. Representative images of the four entire sections are shown. Scale bars, 1mm. (**B**) SDS eluates of s/c tumors formed with sgScramble or sgTGM2-edited PDA cells in wild-type hosts were subjected to SDS-PAGE and immunoblotting with anti-CXCL12 or anti-KRT19 antibodies. (**C**) Sections of s/c tumors formed with sgScramble or sgTGM2-edited PDA cell lines, in wild-type hosts or TGM2-KO hosts, were stained with fluorescent anti-KRT19 and anti-TGM2 antibodies. (**D**) mRNA levels of members of the transglutaminase family in mouse PDA cancer cell organoids are shown (*27*). (**E**) s/c tumors formed with sgScramble or sgTGM2-edited PDA cell lines, in wild-type hosts or TGM2-KO hosts, were stained with fluorescent anti-KRT19 and anti-FXIIIa antibodies. Scale bars, 100 µm.

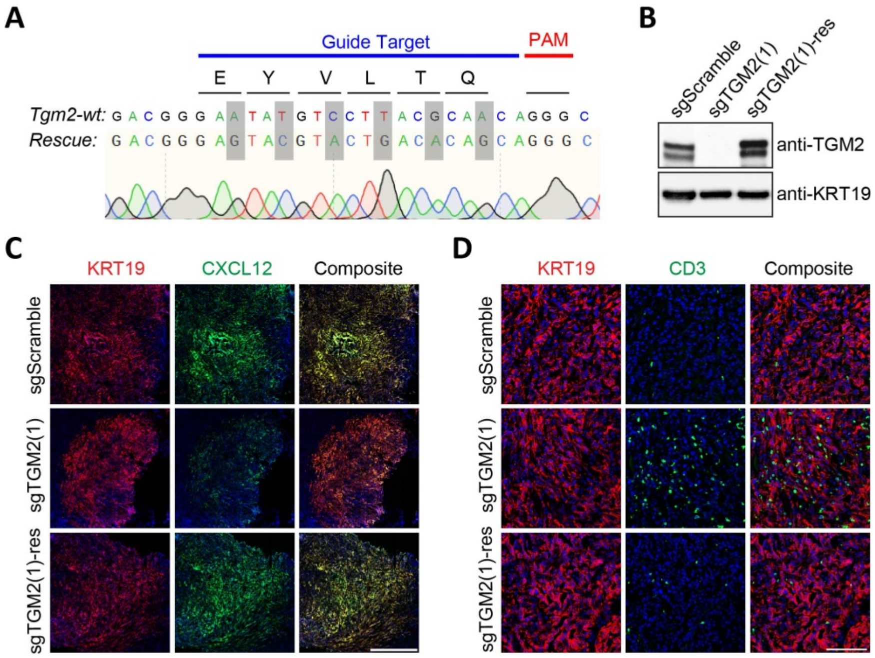

Figure S11: Rescue of TGM2 expression in *Tgm2*-edited PDA cells. (**A**) Sequencing of the TGM2 rescue construct where six synonymous mutations, shown in black boxes, were introduced to prevent the binding of sgTGM2(1). (**B**) PDA cell lysates were subjected to SDS-PAGE and immunoblotting with anti-TGM2 and anti-KRT19 antibodies. (**C**) Tissue sections of s/c tumors formed with the sgScramble PDA cells, sgTGM2-edited PDA cells, and the TGM2-rescue PDA cells were stained with fluorescent antibodies to KRT19 and CXCL12. Scale bar, 0.5 mm. (**D**) Sections from these s/c PDA tumors were also stained with fluorescent antibodies to KRT19 and CD3. Scale bar, 100 μm.

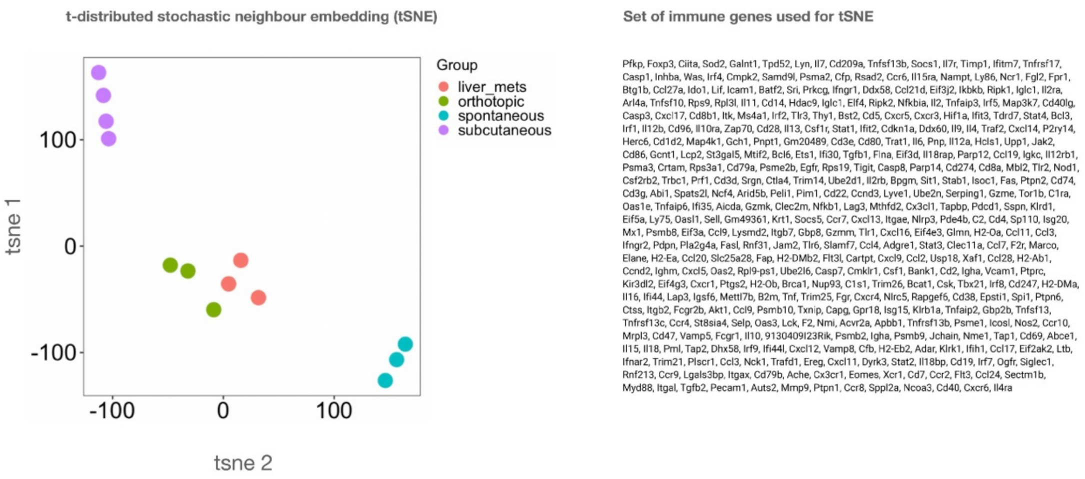

Figure S12: Comparison of immunological microenvironment in different PDA tumor models. *t*-distributed stochastic neighbor embedding (tsne) analysis of immune-related genes in RNAseq data generated from mouse subcutaneous, orthotopic, hepatic metastasis and autochthonous KPC PDA tumors.

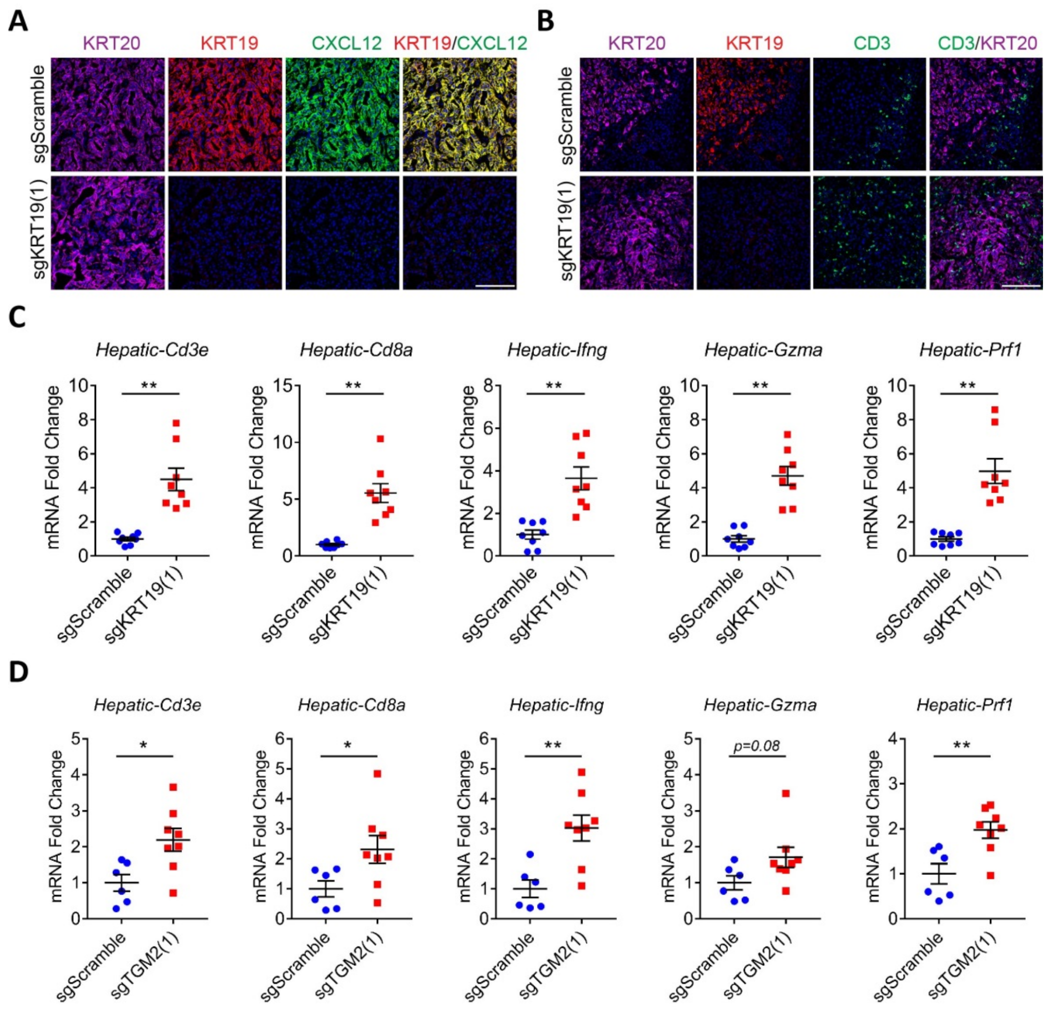

Figure S13: Characterization of *Krt19*- and *Tgm2*- edited mouse PDA hepatic metastases. (**A**-**B**) Sections of the control and *Krt19*-edited PDA metastases were stained with fluorescent antibodies to KRT20, KRT19, CXCL12 and CD3. Scale bars, 100 μm. (**C**-**D**) The mRNA levels in hepatic metastatic tumors formed with sgScramble, sgKRT19 (C) or sgTGM2 (D) edited PDA cells were measured by qPCR and expression of each gene was normalized to that of the control levels, which were assigned a value of 1. Mean ± SEM, n=6 or 8; * p<0.05, ** p<0.01, Student's t test.

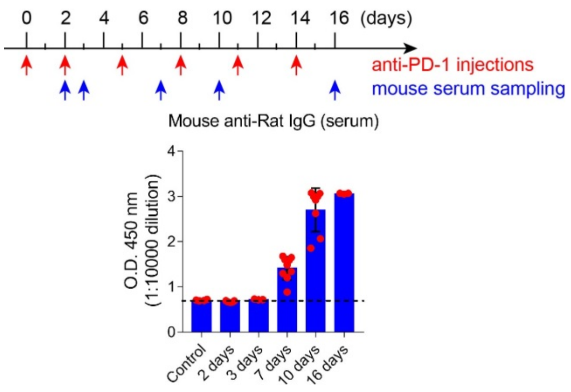

Figure S14: Development of anti-rat IgG in mice receiving rat anti-PD-1 antibody. Mouse anti-rat IgG in blood serum of mice receiving rat anti-mouse PD-1 antibody was measured by means of ELISA. 200 µg rat anti-mouse PD-1 antibody was administered by intra-peritoneal injections at indicated days (red arrows).

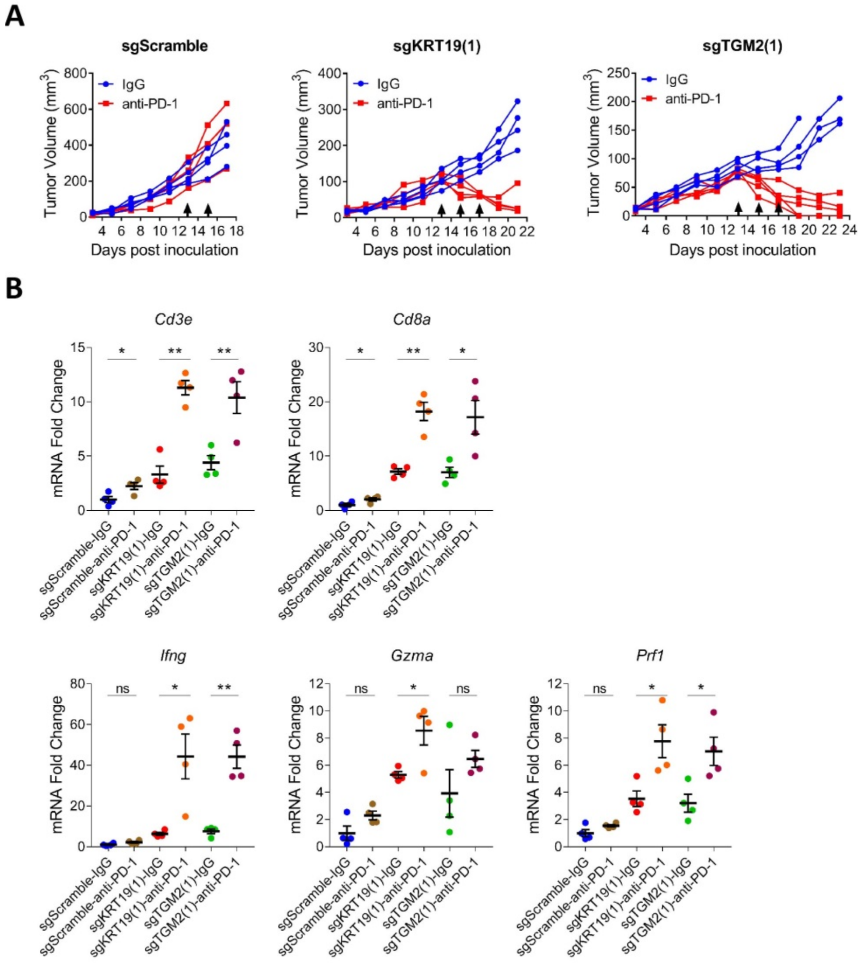

Figure S15: Responses of s/c tumors lacking the CXCL12-KRT19 coating to treatment with anti-PD-1 antibody. (**A**) Mice bearing s/c tumors formed with sgScramble control PDA cells, sgKRT19-edited PDA cells or sgTGM2-edited PDA cells were treated by intra-peritoneal administration of non-immune IgG or anti-PD-1 IgG (arrows), and tumor growth was measured. (**B**) PDA-bearing mice were treated with 200 μg isotype IgG or anti-PD-1 antibody at day 13 and day 15 after cancer cell inoculation. Mice were sacrificed at day 17, and samples of the tumors were obtained. The mRNA levels of each gene in the tumor samples were measured by qPCR. Expression of each gene in sgScramble tumors from mice that had been treated with isotype IgG were normalized to 1. Mean ± SEM, n=4; ns, not significant, * p<0.05, ** p<0.01, Student's t test.

Table S1: Primary antibody list

Antibody	Vendor	Catalog NO.	Reactivity	Application
KRT19-AF568	Abcam	ab203445	Hs, Mm	IF, FC
KRT19-AF647	Abcam	ab192980	Hs, Mm	IF, FC
KRT8-AF647	Abcam	ab192468	Hs, Mm	IF
CXCL12-FITC	R&D	IC350F	Hs, Mm	IF, FC
CD45-BV510	BioLegend	103138	Mm	FC
CD3-AF647	BioLegend	100209	Mm	IF
Tubulin-AF488	Cell Signaling	36234S	Hs, Mm	IF
Tubulin-AF647	Cell Signaling	3624S	Hs, Mm	IF
Mm-anti-CXCL12	R&D	MAB350	Hs, Mm	IF
Rb-anti-CXCL12	Peprotech	500-P87A	Hs, Mm	WB
Rb-Biotin anti-CXCL12	Peprotech	500-P87ABT	Hs, Mm	IP
Rb-anti-CXCL12	LSBio	LS-C48888	Hs, Mm	IP
Rb-anti-KRT19	Abcam	ab76539	Hs	IP
Rb-anti-KRT19	Abcam	ab52625	Hs, Mm	IF, WB
Rt-anti-KRT19	DHSB	TROMA-III	Mm	WB
Rb-anti-KRT20	Abcam	ab230524	Mm	IF
Mm-anti-KRT18	Abcam	ab668	Mm	IF, WB
Rb-anti-KRT8	Abcam	ab53280	Hs, Mm	IF, WB
Sh-anti-TGM2	R&D	AF5418	Mm	IF, WB
Mm-anti-FXIIIa	Abcam	ab1834	Mm	IF
Rb-anti-Actin	Abcam	ab8227	Mm	WB
Rt-anti-CD3	BioLegend	100202	Mm	IF
Rb-IgG Control	Thermo	10500C		IP
Rb-IgG Control	Cell Signaling	3900		IP
Rb-Biotin IgG Control	Abcam	ab200208		IP
Rt-anti-PD-1	BioXcell	BP0273	Mm	PD-1 blocking
Rt-IgG2a Control	BioXcell	BP0089		In vivo
Rt-anti-CD8	BioXcell	BP0061	Mm	T cell depletion
Rt-anti-CD4	BioXcell	BP0003	Mm	T cell depletion
Rt-IgG2b Control	BioXcell	BP0090		In vivo

Table S2: Taqman probes for qPCR and RT-PCR (Thermo)

Gene	Catalog NO.
Tbp	Mm00446973_m1<br>Hs00427620_m1
Cd3e	Mm00599683_m1
Cd8a	Mm01182107_g1
Ifng	Mm01168134_m1
Gzma	Mm01304452_m1
Prf1	Mm00812512_m1
Cxcl12	Hs00171022_m1
